# The higher order auditory cortex is involved in the assignment of affective value to sensory stimuli

**DOI:** 10.1038/ncomms9886

**Published:** 2015-12-01

**Authors:** Anna Grosso, Marco Cambiaghi, Annamaria Renna, Luisella Milano, Giorgio Roberto Merlo, Tiziana Sacco, Benedetto Sacchetti

**Affiliations:** 1 Rita Levi-Montalcini Department of Neuroscience, University of Turin, I-10125 Turin, Italy; 2 Department of Molecular Biotechnology and Health Science, University of Turin, I-10125 Turin, Italy; 3 National Institute of Neuroscience—Turin, I-10125 Turin, Italy

## Abstract

The sensory cortex participates in emotional memory but its role is poorly understood. Here we show that inactivation of the higher order auditory cortex Te2 in rats during early memory consolidation impairs remote first- and second-order fear memories but not the association between two neutral cues. Furthermore, Te2 inactivation prevents changes in the valence of such information. Following the presentation of two auditory cues previously paired with either pleasant or painful stimuli, a large percentage of cells responds to both experiences but also a small fraction of neurons responds exclusively to one of them. The latter type of neurons signals the valence rather than the salience or the motor responses associated with the stimuli, and reflects selective associative processes. Pharmacogenetic silencing of memory-activated neurons causes amnesia. Thus, Te2 represents a crucial node for the assignment of the affective value to sensory stimuli and for the storage of such information.

## Abstract (web summary)

The auditory cortex Te2 represents a key node for the assignment of the affective value to sensory stimuli in rodents. Using pharmacogenetic manipulations, this study shows that in Te2 there are neurons which respond to the emotional valence of sounds and their inactivation impairs emotional memories retrieval.

## Graphical abstract

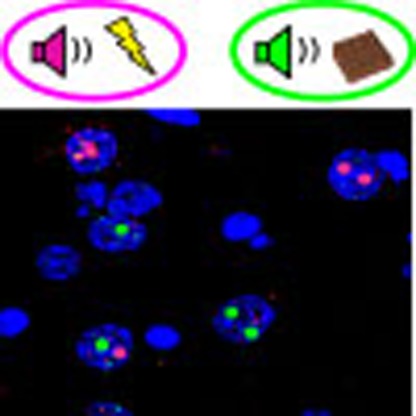

Encoding and retrieving memories of past emotional events are key functions of the brain. How and where in the brain sensory stimuli are linked to their affective charges are, however, far from being defined. The amygdala and other nuclei, such as the nucleus accumbens and the ventral tegmental area, play a key role in this process^1^,^2^,^3^,^4^,^5^,^6^. In addition, starting in the mid-1980s, Scheich and colleagues^7^,^8^ and Weinberger *et al*.^9^,^10^ discovered learning-evoked changes in the auditory cortex. Since then, an increasing number of studies has emphasized that the auditory cortex, whether primary or higher order, is critically involved in associative and higher cognitive processes. Thus, the initial view that the auditory cortex is mostly a stimuli’s analyser that transmits sensory information to the amygdala^1^, especially for more complex auditory stimuli, is changing in favour of more complex roles^9^,^10^,^11^.

In support of this notion, we recently identified higher order components of the sensory cortices, in particular the temporal auditory cortex Te2, that are essential for the storage of remote, but not recent, fear memories^12^. These cortices are anatomically and functionally connected to the primary cortices and to subcortical nuclei^13^,^14^,^15^, such as the amygdala and the nucleus accumbens. Therefore, these higher order cortices may represent sites where sensory and emotional information converge, so that sensory stimuli become linked to their emotional values. In the present study, we sought to address this question. We found that the Te2 is involved, shortly after training, in the formation of remote fear memories obtained through both first- and second-order conditioning protocols, while the same area is not necessary for pairing two neutral stimuli. Moreover, during the recall of remote fear memories, a small fraction of neurons in Te2 is activated by the particular valence, but not by the salience previously associated with the sounds. These neurons reflects selective associative processes linking sensory cues to emotional representations. Pharmacogenetic silencing of Te2 neurons activated by fear memories results in amnesia only for fear, but not for appetitive, information. We interpret these data to mean that higher components of the auditory cortex are necessary for the encoding and storage of the affective meaning that has been associated with sensory stimuli during an experience.

## Results

### Te2 is involved in the formation of remote fear memories

**Figure 1:**
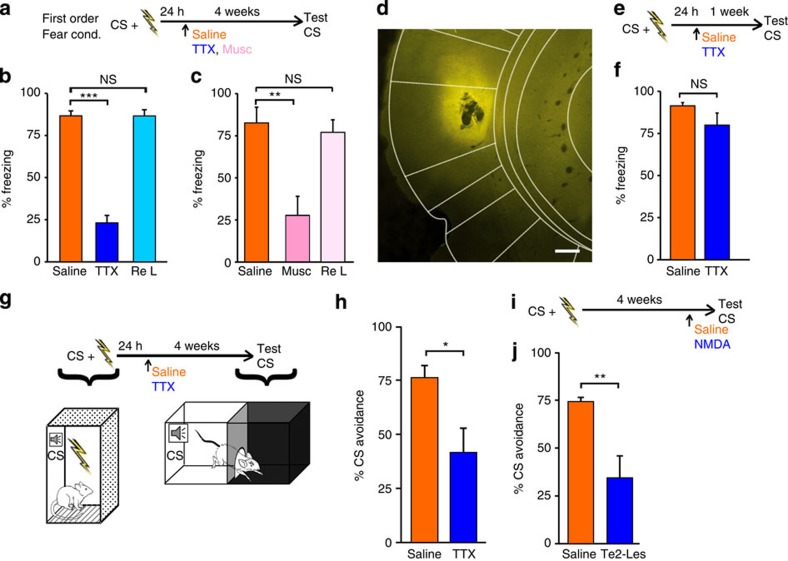
Te2 is necessary for the early formation and the subsequent storage of fear memories, independent of the motor responses paired to learned fear stimuli. (**a**) Experimental design for remote memory testing after first-order fear conditioning; Musc, muscimol; TTX, tetrodotoxin. (**b**) Freezing was measured during the CS presentation (CS) in saline-treated (Saline, *n*=13) and TTX-treated (*n*=13) rats. TTX-treated animals displayed amnesic effects (Student's *t*-test*, t*_24_=11.80, *P*<0.001). Amnesic rats were able to form and retain new memories (Re L, *t*_12_=1.6, NS). (**c**) Muscimol administration induced amnesia (Saline, *n*=8; Musc, *n*=10; *t*_16_= 3.3, *P*<0.01) but did not block the acquisition of new recent memories and their retention (Re L, *t*_16_=0.48, NS). (**d**) Representative fluorescent micrograph showing injection of muscimol-BODIPY TMR-X conjugate into Te2. Scale bar, 200 μm. (**e**) Experimental design for recent memory testing after first-order fear conditioning. (**f**) Freezing response was similar in TTX-treated (*n*=14) and saline-treated (*n*=14) animals (*t*_26_=1.5, NS). (**g**) Experimental design for avoidance response testing, using a two-chamber light–dark box. (**h**) The CS avoidance response of TTX-injected rats (*n*=8) was significantly lower with respect to saline-treated rats (*n*=8) (*t*_14_=−2.760, *P*<0.05). (**i**) Experimental design to test the effect of Te2 lesioning on retrieval of remote memories. (**j**) NMDA-lesioned rats (Te2-Les, *n*=8) did not display avoidance behavior towards the CS (*n*=8; *t*_14_= −3.264, *P*<0.01). *P<0.05; **P<0.01, ***P<0.001; NS, not significant. Data shown are mean and s.e.m.

Our previous study showed that irreversible lesions of Te2, 1 day after rats were trained to associate a tone (conditioned stimulus, CS) to a foot shock (unconditioned stimulus, US), did not impair the recall of recent fear memories^12^. Here we investigated if at this time point Te2 is recruited for the early consolidation of remote fear memories (Fig. 1a). To block memory consolidation processes without interfering with the acquisition or retrieval phases, we employed a reversible inactivation technique. Rats were trained to associate a tone (CS) with an inescapable foot shock (US) and, 1 day later, Te2 was inactivated by injection of tetrodotoxin (TTX), a sodium channel blocker^16^,^17^. Memory retention was assessed 1 month later by measuring freezing in response to the CS. Freezing was significantly lower in TTX-treated animals than in sham-treated control rats, indicating that Te2 is recruited early after training to form remote memories (Fig. 1b). When the TTX-treated rats were retrained in the fear conditioning protocol and retested after 1 day, they showed no impairment (Fig. 1b), indicating that they retained the ability to learn new memories.

Because TTX blocks both local neurons and fibres of passage, we repeated the experiments using muscimol, a GABA_A_ receptor agonist that reversibly blocks only local neurons^18^. When tested 1 month later, muscimol-treated rats displayed amnesia (Fig. 1c,d). Because amnesia was observed 1 month after the administration of TTX or muscimol, it cannot be ascribed to the drugs’ functional impairment of Te2 at the moment of the test, and Nissl staining confirmed the absence of any permanent tissue damage (Supplementary Fig. 1). We also observed similar amnesic effects in animals treated with TTX in the secondary visual cortex shortly after conditioning to a visual CS (Supplementary Fig. 2), suggesting that the early cortical involvement in the emotional memory storage extends beyond the auditory system.

In another experimental group, TTX was injected 1 day after training, as before, but memory retention was tested after only 1 week (Fig. 1e). Freezing response in TTX-treated animals was similar to that of sham-treated controls (Fig. 1f). These data confirm that Te2 is not necessary for the formation of recent memories, as we already showed^12^.

Altogether, these findings indicate that Te2 activity is required early after training for the formation of remote, but not recent, fear memories. Because remote, but not recent, hippocampal-dependent memories also require cortical processes early after training to form^17^, this early engagement of cortical structures may be a general rule.

Recent studies reported that motor activity influences auditory processes in the primary auditory cortex^19^,^20^,^21^,^22^. Thus, the auditory cortex, including Te2, may be part of a sensorimotor system that links auditory stimuli to specific motor responses. To determine whether the reduced freezing response in TTX-treated animals was the expression of an impaired association between a given motor response and the CS, we tested memory retention using a light–dark box (Fig. 1g). Rats were trained to associate the CS to an inescapable foot shock so as to elicit conditioned freezing response, as before. One month later, animals were put in the light–dark box and the CS began when the animal was exploring the lighted chamber; the animal could seek refuge in the dark chamber to avoid the CS. Such avoidance behaviour is an innate response to danger, not a conditioned response as a result of pairing with the CS^23^. TTX-treated rats spent significantly less time in the dark (lower avoidance response) than did sham-treated controls (Fig. 1h). These results indicate that Te2 inactivation during early consolidation impairs innate reactions to a learned threat, and suggest that the role of Te2 in emotional learning goes beyond the association of sounds to specific motor reactions.

In a similar experiment, rats underwent fear conditioning followed, 1 month later, by irreversible Te2 lesioning with *N*-methyl-D-aspartic acid (NMDA)^12^ (Fig. 1i). NMDA-treated rats had a significantly lower avoidance response than did sham-treated controls (Fig. 1j), indicating that the role of Te2 in remote memory storage/retrieval is not limited to linking CSs to specific motor responses.

### Te2 associates sensory cues with their affective properties

**Figure 2:**
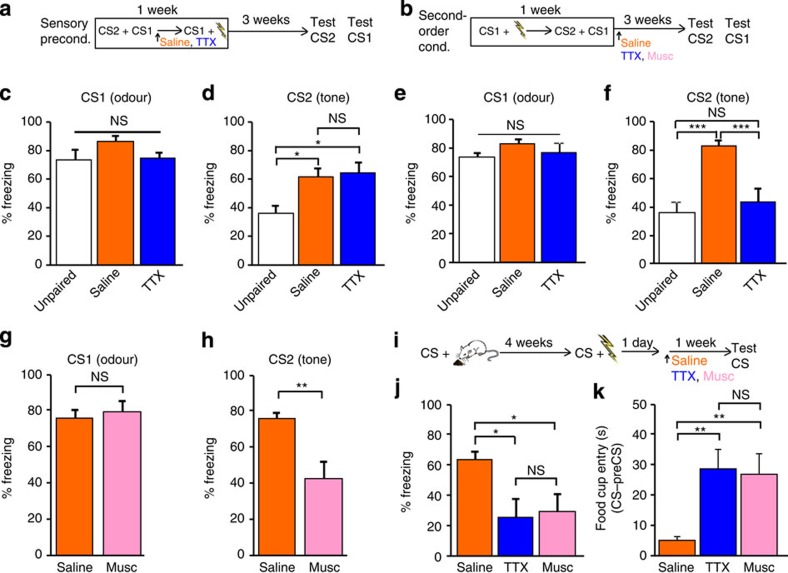
Te2 encodes the emotional valence of sensory stimuli. (**a**) Experimental design for sensory preconditioning. (**b**) Experimental design for second-order conditioning. CS1, odour; CS2, tone. TTX, tetrodotoxin; Musc, muscimol. (**c**) In the sensory preconditioning paradigm, freezing response to the CS1 was high and similar among the Unpaired (*n*=8), saline-treated (Saline, *n*=11) and TTX-treated animals (*n*=11); one-way ANOVA (F_(2,27)_=1.99, *P*>0.05). (**d**) The freezing response to CS2 was substantially lower in the Unpaired group but remained high in animals exposed to the CS1–CS2 pairing, both saline-treated and TTX-treated groups (one-way ANOVA F_(2,27)_=4.84, *P*<0.05; Newman–Keuls test *P*<0.01). (**e**) After second-order conditioning, the freezing response to CS1 was similar among the Unpaired (*n*=8), saline-treated (*n*=11) and TTX-treated animals (*n*=11); one-way ANOVA (F_(2,27)_=0.62, NS). (**f**) In contrast, TTX-injected animals were impaired in the memory retention to the auditory CS2 (F_(2,27)_=11,58, *P*<0.001). (**g**) Similar freezing response to CS1 in saline-treated (*n*=8) and muscimol-treated (Musc, *n*=7) animals (Student's *t*-test*, t*_13_=−0.47, NS). (**h**) Muscimol-treated animals displayed amnesia to CS2 (*t*_13_= 3.26, *P*<0.01). (**i**) The appetitive-to-fear conditioning protocol. (**j**) Freezing responses to CS after valence switching were less in TTX-treated animals (*n*=9) and muscimol-treated animals (*n*=8) than in the saline-treated group (*n*=10; F_(2,24)_=4.43, *P*<0.05). (**k**) The total time animals spent searching for an award (that is, food cup entry time; CS, preCS in seconds) was similar between TTX- and muscimol-treated rats, but significantly higher than that of the saline-treated group (F_(2,24)_=6.17, *P*<0.01; Newman–Keuls test, *P*<0.01). *P<0.05; **P<0.01, ***P<0.001; NS, not significant. Data shown are mean and s.e.m.

Our findings raise the important question of what information about the CS is encoded in Te2. Emotional learning involves several processes: the elaboration and the subsequent memorization of the sensory features of CS and US (‘perceptual learning’), associative processes linking sensory stimuli (‘S-S learning’), and finally the association between the CS and the value and/or the responses of the US with which the CS has been paired. Many learning paradigms, such as first-order fear or appetitive conditioning, engage these processes simultaneously and thus do not allow them to be studied separately. We therefore opted for another approach to circumvent these obstacles. We used two related learning paradigms—sensory preconditioning and second-order fear conditioning. In sensory preconditioning, animals are trained to associate two distinct neutral sensory stimuli (CS1–CS2 pairing) and then are conditioned to associate CS1 to an aversive US (Fig. 2a). In second-order fear conditioning, animals are first trained to associate CS1 with the aversive US, and then are exposed to CS2–CS1 pairing^24^,^25^,^26^ (Fig. 2b). During CS2–CS1 pairing in sensory preconditioning, animals learn to associate two distinct neutral sensory stimuli; in second-order fear conditioning, they learn to associate a CS with the emotional response elicited by another CS^24^,^25^,^26^. By inactivating Te2 shortly after CS2–CS1 pairing in both paradigms and then testing memory retention after 1 month, we aimed to test whether Te2 was involved in perceptual learning, S-S learning, or the association of a CS with an emotional response.

In the sensory preconditioning paradigm, freezing responses to CS1 (almond odour) were high and similar between TTX-treated animals and two control groups, namely saline-treated animals exposed to CS1–CS2 pairing and animals exposed to CS2 and CS1 unpaired in time (Fig. 2c). When tested with CS2 (tone), both the TTX- and saline-treated animals maintained their strong freezing responses, which, however, were significantly lower in the unpaired animals (Fig. 2d). In the second-order fear conditioning paradigm, similarly high freezing responses to CS1 were observed in all three groups (Fig. 2e), whereas TTX treatment had a significant amnesic effect towards CS2 (Fig. 2f). Similarly, muscimol-treated rats displayed amnesia to CS2 (Fig. 2h) but not CS1 (Fig. 2g, Supplementary Fig. 3). Thus, Te2 activity is required to form the association between sensory cues and their affective properties, but it is not required for perceptual learning or S–S learning.

An alternative explanation for the amnesia observed in second-order fear conditioning is that Te2 is necessary for storing memories of a CS that has acquired a behavioural salience, irrespective of the associated emotional aspect. Emotional experiences are characterized by two primary dimensions: intensity, ranging from weak to strong, and valence, ranging from negative to positive^27^,^28^. To test whether Te2 inactivation affected the valence associated with a CS, we used an appetitive-to-fear conditioning paradigm. Rats were exposed to an auditory CS paired with a pleasant US (food reward) and, 4 weeks later, the same CS was paired with an aversive US, reversing its affective value; Te2 was reversibly inactivated 1 day later (Fig. 2i). One week later, a significantly lower freezing response was observed in the treated animals than in controls (Fig. 2j). Rather, the treated animals retained the conditioned response learned before valence switching (Fig. 2k). Thus, Te2 silencing prevented the change in emotional valence of a remote memory, without affecting the memory of such CSs associated with their previously acquired emotional value. Given that memory retention was tested 1 week later, these results are at odds with those shown in Fig. 1, where memory tested 1 week after training was unaffected by Te2 manipulations. The difference can be explained by the fact that, in the present paradigm, the original memory was formed 1 month before the change in emotional valence and therefore it became dependent on the cortex.

The amnesia observed in the appetitive-to-fear conditioning experiments cannot be due to an inability to process or encode CS features, because this information was acquired several weeks before Te2 inactivation. Indeed, Te2 inactivation did not disrupt memory of the CS, as shown by the high level of appetitive memory towards the CS. Furthermore, because Te2 remained intact for 24 h after memory recall, the amnesia cannot be due to a temporary inability to recall the CS representation or to transmit this information to other sites. We can also rule out an interference with appetitive memory reconsolidation processes, which terminate within a few hours after appetitive memory recall^29^. We conclude that amnesia in Te2-lesioned animals is due to a specific interference with the change in the value assigned to the CS. Thus, Te2 participates in the encoding of the valence of perceived stimuli and to the subsequent changes of such information.

### Te2 is necessary for remote appetitive memories

**Figure 3:**
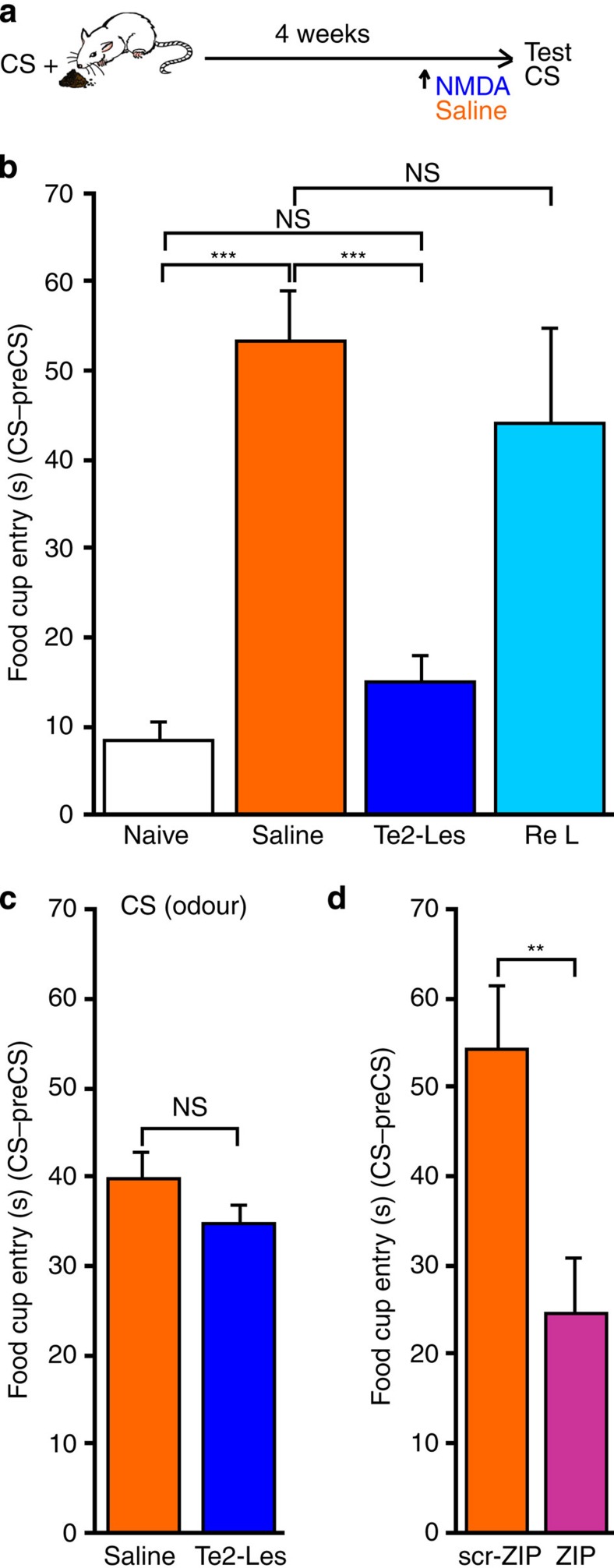
Higher order sensory cortices are needed for the storage of remote appetitive memories. (**a**) Experimental design for first-order appetitive conditioning. (**b**) Time spent in the food cup searching for a reward in response to the CS, for naive unconditioned rats (Naive, *n*=11), saline-treated rats (Saline, *n*=11) and NMDA-treated rats (Te2-Les, *n*=10). One-way ANOVA (F_(2,29)_=36.45, *P*<0.001) and Newman–Keuls test indicated that Te2-lesioned animals differed from sham-treated rats (*P*<0.001) but not from naïve unconditioned rats (*P*>0.05, NS). After a second round of training, NMDA-treated animals relearned the CS–US association (Re L), spending a similar amount of time in the food cup as sham-treated animals (Student’s *t*_19_=0.77, *P*>0.05, NS). (**c**) Animals with Te2 lesions were able to retrieve remote appetitive memories of an olfactory CS (*t*_14_=1.49, *P*>0.05, NS). (**d**) Animals (*n*=9) that received zeta inhibitory peptide (ZIP), which inactivates protein kinase Mzeta, in Te2 also spent less time searching for a reward than animals treated with the inactive scrambled peptide (scr-ZIP, *n*=8; t_15_=−3.14, *P*<0.01). **P<0.01, ***P<0.001; NS, not significant. Data shown are mean and s.e.m.

Given that in the previous experiment Te2 blockade had no effect on remote appetitive memories (Fig. 2k), we tested whether Te2 is involved in the storage/retrieval of these memories (Fig. 3a). Rats were trained to associate an auditory CS with a pleasant US. After one month, the Te2 was irreversibly lesioned with NMDA (Supplementary Fig. 4). NMDA-treated animals displayed a significantly lower conditioned response than did sham-treated animals, but were able to relearn the CS–US association (Fig. 3b). Remarkably, Te2 lesions did not affect appetitive memories of an olfactory CS (Fig. 3c), thus supporting the view that each sensory cortex is selectively involved in the storage/retrieval of remote appetitive memories of the sensory CSs that it elaborates^12^. The latter results rule out the possibility that the amnesia was due to an impairment of emotional or motor responses.

Because irreversible lesions can cause secondary neuronal loss in the structures to which the sensory cortex projects, the amnesia observed here may have been due to such a secondary impairment. Therefore, we repeated the experiment using the zeta inhibitory peptide (ZIP), which inactivates several cellular processes, such as those involving protein kinase Mzeta (PKMz)^30^,^31^, and causes amnesia even after the peptide has been eliminated, allowing memory retention to be tested with the brain structure functionally intact^12^,^30^. Rats were trained in the appetitive conditioning protocol and, one month later, received ZIP in Te2. Memory was tested two days later, that is, when the peptide had been eliminated. ZIP-treated animals displayed amnesia (Fig. 3d).

Similar results were obtained in animals conditioned to visual CSs and lesioned in the secondary visual cortex (Supplementary Fig. 5). These data indicate that appetitive memories are stored, at least in part, in a distributed cortical network formed by higher order sensory cortices. Collectively, our behavioural findings suggest that higher order sensory cortices are crucial nodes for the assignment of negative and positive affective values to sensory stimuli and for the storage/retrieval of such information.

### Te2 neurons for pleasant or unpleasant values

We then investigated the molecular mechanisms by which Te2 stores the affective value of sensory stimuli. To this aim, we used the cellular compartment analysis of temporal activity by capture antibody-targeted fluorescent *in situ* hybridization (catFISH) technique to monitor the expression of two immediate early genes, *Arc/Arg 3.1* (*Arc*) and *Homer 1a* (*H1a*), that have distinct temporal patterns of expression in neurons: *Arc* messenger RNA (mRNA) is detectable in the nucleus 5–8 min after a salient event while *H1a* mRNA appears 25–30 min later^32^,^33^. Thus, in animals tested for two behaviours separated by an interval of 20 min, catFISH reveals the first behaviour as nuclear *H1a* and the second behaviour as nuclear *Arc* expression. Expression of only one gene indicates selectivity for one of the two events, while double labelling means a neuron was engaged by both behavioural epochs. This analysis was focused on layer 2/3 because previous studies showed a maximal activation at this cortical level following memory recall^12^,^17^.

**Figure 4:**
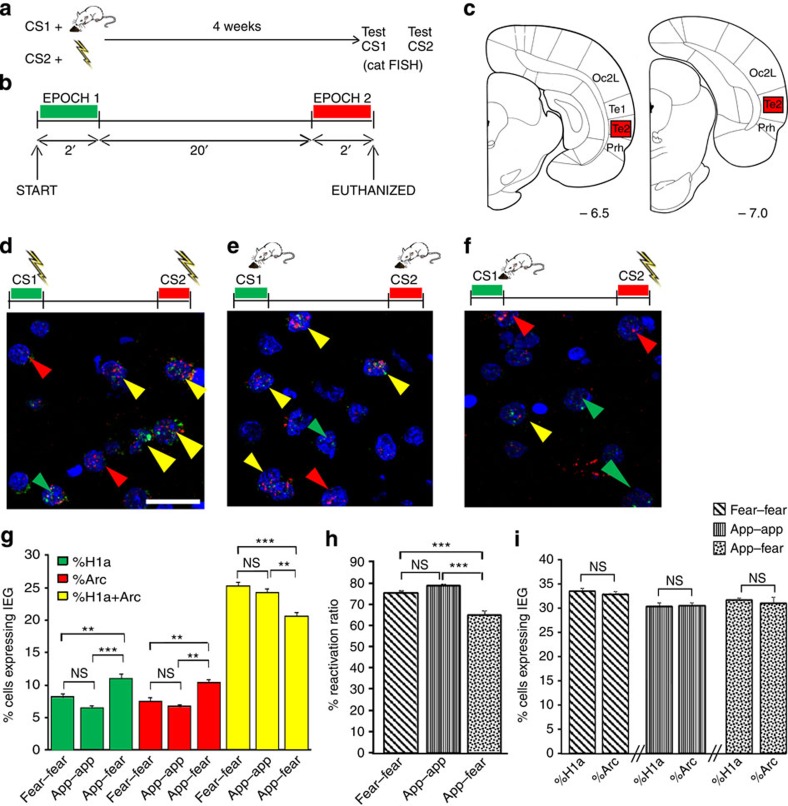
Te2 activity following the recall of aversive or appetitive remote memories. (**a**) Experimental design for appetitive–fear conditioning. Rats were trained to associate two different auditory stimuli (CSs) to opposite emotional experiences. One month after training, rats were tested for memory retention to CS1 and, after 20 min, to CS2. Immediately after testing, fluorescent in situ hybridization to detect *Arc/Arg 3.1* (*Arc*) and *Homer 1a* (*H1a*) expression (catFISH method) was performed. CS1 and CS2, conditioned stimuli 1 and 2. (**b**) Time course for remote memory testing 1 month after training. Conditioned responses to CS1 were measured in a 2-min test and then, after a 20-min pause, responses to CS2 were measured. (**c**) Two cortical sections across Te2 cortex were analysed. Plates adapted from Zilles^53^. Oc2L, secondary occipital visual cortex; Prh, perirhinal cortex; Te1 and Te2, primary and secondary auditory cortices. (**d**) Representative fluorescent photomicrographs showing neuronal nuclei that expressed primarily *H1a* mRNA (green arrowheads) or *Arc* mRNA (red), or that expressed both (yellow) in fear–fear (**d**), appetitive-appetitive (app-app), (**e**) and appetitive–fear (**f**) groups. Scale bar, 20 μm. (**g**) Percentage of cells expressing immediately early genes (IEG) (only *H1a*, only *Arc*, or both genes) in fear–fear conditioned rats (*n*=9), appetitive–appetitive conditioned rats (*n*=6), and appetitive–fear conditioned rats (*n*=8). The percentages of neurons expressing only *H1a* (F_(2,20)_=16.61, *P*<0.001) and only *Arc* (F_(2,20)_=12.51, *P*<0.001) were significantly higher in the appetitive–fear group than in either the fear–fear or appetitive-appetitive group, with a corresponding reduction in doubly labelled cells (F_(2,20)_=14.53, *P*<0.001). (**h**) The reactivation ratio, calculated as the percentage of cells expressing both *Arc* and *H1a* divided by the number of cells expressing *H1a*, was significantly less in the appetitive–fear group than in the fear–fear and appetitive-appetitive groups (F_(2,20)_=19.78, *P*<0.001). (**i**) In each group, the percentage of neurons activated during epoch 1 (*H1a*-positive cells) was similar to that of *Arc* (fear–fear, *t*_16_=0.69, NS; app–app, *t*_10_=0.26, NS; appetitive–fear, *t*_14_=−0.83, NS). **P<0.01, ***P<0.001; NS, not significant.

We studied three conditioning paradigms (Fig. 4a,b, Supplementary Fig. 6). In fear–fear conditioning, rats were trained to associate two different auditory CSs to the same aversive US (foot shock); when tested for memory retention 4 weeks later, both CSs elicited a conditioned freezing response (Supplementary Fig. 7a,b). In appetitive–appetitive conditioning, the two CSs were paired to an appetitive US (food reward), and they prompted conditioned appetitive responses in the animals (Supplementary Fig. 7c,d). In appetitive–fear conditioning, an auditory CS1 was paired with a pleasant US, while CS2 was associated with an aversive US; the subsequent presentation of CS1 elicited a conditioned appetitive response while CS2 caused fear behaviour (Supplementary Fig. 7b,c). When Te2 neurons were stained for *Arc* and *H1a* mRNA immediately after the testing, we observed that most neurons were doubly labelled in the fear–fear conditioning group (Fig. 4d,g), suggesting that two memories endowed with similar emotional content activated the same neuronal population in Te2. Similar results were observed with appetitive–appetitive conditioning (Fig. 4e,g). In contrast, in appetitive–fear conditioning, the percentages of cells stained positively to either *H1a* or *Arc* were significantly higher than in the other conditioning protocols, and there was a corresponding decrease in doubly labelled cells (Fig. 4f,g).

To further depict the differential activation of Te2 neurons after the behavioural procedures, the percentage of doubly labelled cells was divided by the percentage of *H1a*-positive cells. A reactivation ratio near 100% indicates that the two behaviours activate the same neuronal populations, while lower values indicate that distinct neurons are involved^33^,^34^. This analysis revealed that the appetitive–fear conditioning group had a significantly lower reactivation ratio than did the other groups (Fig. 4h). To ensure that the effects we observed were due to the behavioural manipulation and were not secondary to incidental differences in the expression of *Arc* or *H1a*, we compared within each behavioural group the percentage of neurons activated during epoch 1 (*H1a*-positive cells) to the percentage of neurons positive for *Arc*^33^. No significant differences were detected in any conditioning protocol (Fig. 4i).

These data on *Arc* and *H1a* staining were collected from two distinct cortical subregions in Te2 (Fig. 4c). To investigate if the contrasting emotional memories had a preferential anatomical localization, we analysed the data from the two cortical regions separately but found no significant differences (Supplementary Fig. 8), that is, the two emotional memories engage neurons broadly distributed across Te2.

Altogether, these results suggest that, in Te2, a large population of neurons responds to both affective experiences, while there also exists a small fraction of neurons that exclusively responds to one of the two learned emotional associations.

### Te2 neurons encode the learned valence of stimuli

**Figure 5:**
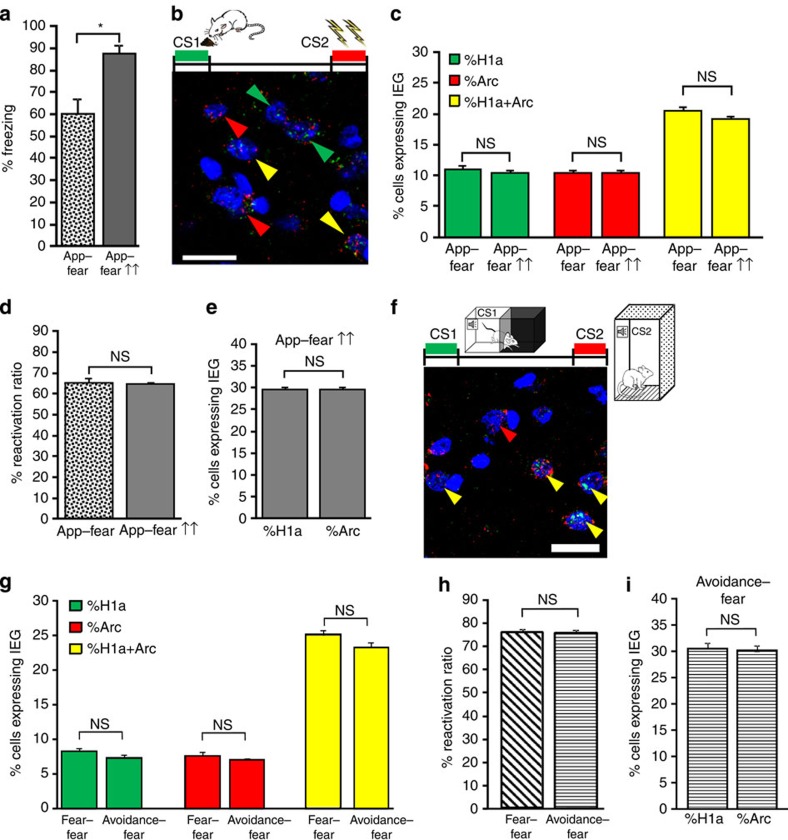
Te2 neurons encode the memory valence but not the salience nor the different behavioural response. (**a**) A more intense aversive memory was obtained by increasing the strength of aversive conditioning (app–fear↑↑, *n*=4). Freezing response was higher in this appetitive–fear group than in the previous appetitive–fear experiment (*t*_10_= −2.56, *P*<0.05). (**b**) Representative fluorescent photomicrograph of cortical neurons in animals conditioned to stronger foot shock stimuli. Scale bar, 20 μm. CS1 and CS2, conditioned stimuli 1 and 2. (**c**) The percentages of cells expressing only *H1a* (*t*_10_=−0.58, NS), only *Arc* (*t*_10_= 0.15, NS) or both *H1a* and *Arc* (*t*_10_=−1.43, NS) were similar between stronger (app–fear↑↑) and weaker (app–fear) fear-conditioned animals. (**d**) Similarly, there were no significant differences between groups in the reactivation ratio (*t*_10_=−0.15, NS). (**e**) In the animals conditioned with stronger painful stimuli, the percentage of *H1a*-positive cells was similar to that of *Arc*-positive cells (t_6_=−0.01, NS). (**f**) Representative fluorescent photomicrograph showing *H1a* (green arrows), *Arc* (red arrows) or doubly (yellow arrows) labelled neurons in the avoidance-fear group (*n*=5). Scale bar, 20 μm. (**g**) The percentages of neurons expressing *H1a* (*t*_12_=−1.16, NS), *Arc* (*t*_12_=−0.6, NS) or both (*t*_12_=−1.87, NS) were similar in the avoidance-fear and fear–fear groups. (**h**) No significant differences between groups in the reactivation ratio were noted (*t*_12_=0.48, NS). (**i**) In the avoidance–fear group, the percentage of *H1a*-positive cells was similar to that of *Arc*-positive cells (*t*_8_=−0.24, NS). * P<0.05; NS, not significant. All values are mean and s.e.m.

Our findings suggest that Te2 contains neurons whose activity signals the affective value assigned to auditory stimuli. Alternatively, however, it is possible that these distinct neural populations reflect the different levels of intensity (for example, the salience) of the emotional experiences. To test this possibility, we devised an experiment in which the salience, but not the valence, of the two CSs was modulated. Animals were conditioned to hedonic and aversive stimuli as in the appetitive–fear conditioning protocol, but this time they received a stronger painful US, so as to elicit a stronger (for example, more salient) aversive memory. Testing of remote memory retention revealed a significantly higher freezing response than when animals were conditioned to the mild US in the appetitive–fear conditioning protocol (Fig. 5a). In these rats, however, the percentages of neurons that responded separately to appetitive and aversive CSs were similar to those obtained in the previous experiment (Fig. 5b-e). Hence, changes in the salience of the two CSs did not modify the percentages of neurons responding separately to the two events.

We then investigated whether the two neuronal populations reflect different motor responses paired to the two CSs. Rats were trained to associate one CS to an inescapable footshock, so as to induce freezing behaviour, while another CS was paired with an escapable footshock, so as to induce an avoidance behaviour (Supplementary Fig. 9). Subsequent catFISH analysis revealed that the percentages of cells stained positively to either *H1a* or *Arc* were similar between the fear–fear and fear–avoidance conditioning groups (Fig. 5f–i).

Altogether, these data suggest that the activity of Te2 neurons reflects neither the salience nor the different motor responses paired to the CSs, further supporting the view that Te2 encodes the valence (aversive or positive quality) of a memory.

### Te2 neurons reflect associative processes

**Figure 6:**
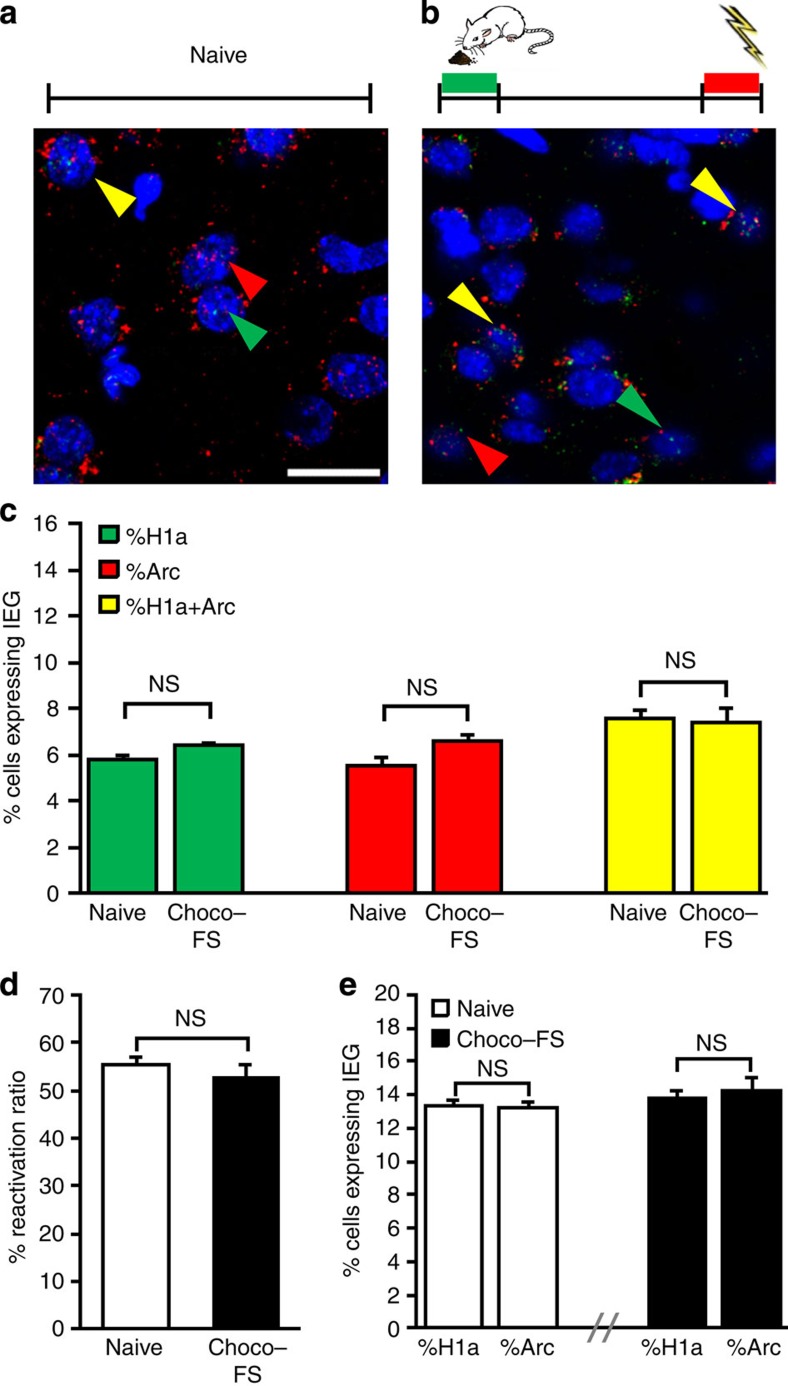
Neuronal activity in Te2 signals the affective valence acquired by auditory CSs but is unchanged following unconditioned affective stimulation. (**a**,**b**) Representative fluorescent photomicrographs of *H1a* (green arrows), *Arc* (red arrows) and doubly (yellow arrows) labelled neurons in naive, untreated animals (*n*=6) (**a**) and in animals that received pleasant (chocolate-flavoured food) and aversive (foot shock) stimuli unpaired with explicit sensory cues (choco-FS, *n*=6). CS1 and CS2, conditioned stimuli 1 and 2. (**b**) Scale bar, 20 μm. (**c**) No significant differences in the immediately early genes (IEG) expression were detected between naive animals and those that received unconditioned emotional stimuli: *H1a* (*t*_10_=−1.93, NS), *Arc* (*t*_10_= 0.34, NS), and both *H1a* and *Arc* (*t*_10_=0.27, NS). (**d**) The reactivation ratio was also similar between the groups (*t*_10_=0.83, NS). (**e**) The percentage of *H1a*-positive cells was similar to that of *Arc*-positive cells, for both the naïve (*t*_10_=−0.36, NS) and the choco-FS (*t*_10_=0.41, NS) rats. NS, not significant. All data are mean and s.e.m.

The differential activity of the Te2 neurons could reflect a bias imparted by the appetitive or aversive behaviours expressed during the tests of memory retention. Therefore, we asked whether these cortical neurons respond to innate emotional stimuli or, alternatively, whether they mainly encode the learned affective value assigned to sensory stimuli during emotional experiences. To this aim, painful stimuli and food rewards were presented in the absence of any explicit sensory cues, so as to prevent associative processes linking emotional experiences to sensory stimuli^35^,^36^. The percentages of cortical cells expressing only *H1a*, only *Arc*, or both *H1a* and *Arc* did not differ between these animals and naive animals that did not receive any behavioural training (Fig. 6a–c). The reactivation ratios were also similar between naive and treated rats (Fig. 6d). Thus, unconditioned emotional experiences by themselves do not activate distinct neurons in Te2, ruling out the possibility that the activity of such neurons reflects emotional or motor responses. Hence, the differential activity of neurons described in the previous experiment (see Fig. 4c–g) is likely to have resulted from associative processes linking sensory cues with emotional representations. We conclude that these neurons form an enduring memory code for the affective valence assigned to CSs and can be considered ‘associative value-coding’ neurons.

### Value-coding neurons in the lateral amygdala

**Figure 7:**
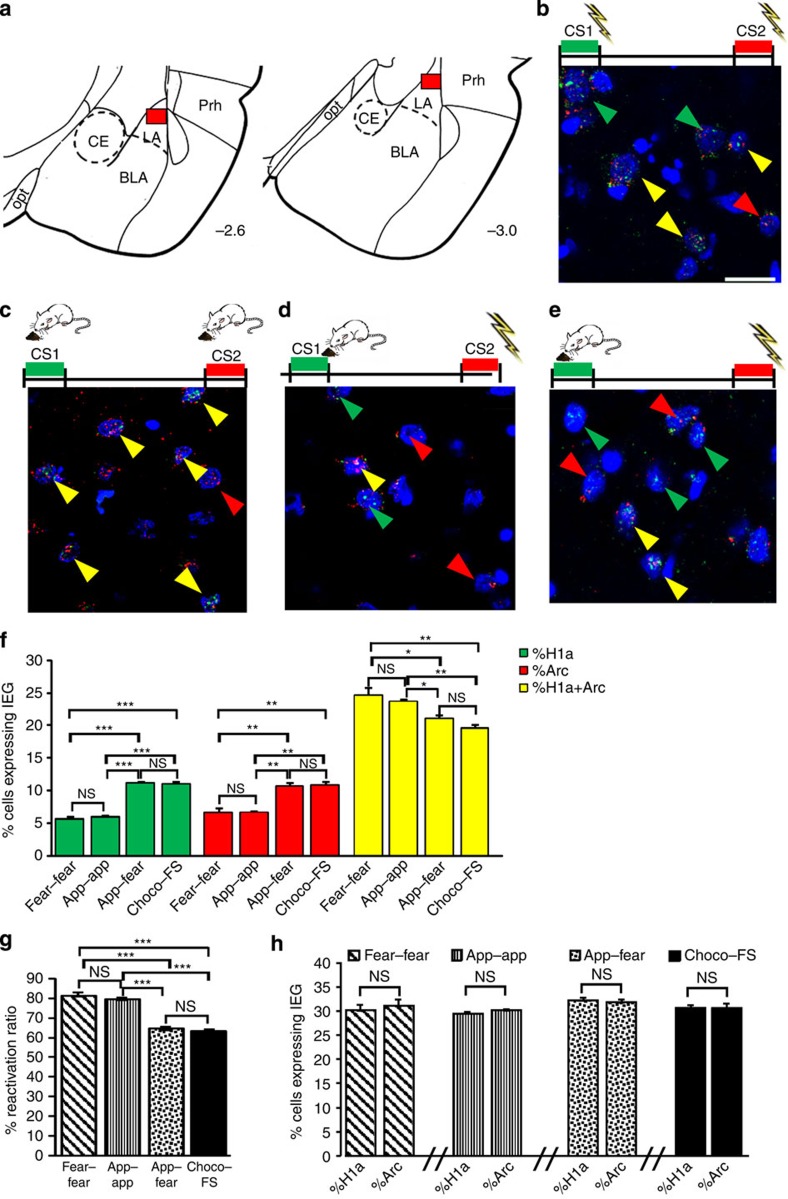
Anatomically distinct neurons in the lateral amygdala reflect emotionally charged unconditioned and learned stimuli. (**a**) catFISH analysis was performed in the lateral region of the amygdala (lateral amygdala). Plates adapted from the Zilles atlas^53^. BLA, basal lateral amygdala; CE, central amygdala; Opt, optic tract; PRh, perirhinal cortex. (**b**,**e**) Representative fluorescent photomicrographs showing neurons expressing *H1a* (green arrows) or *Arc* (red arrows) or both (yellow arrows) in fear–fear (*n*=5) (**b**), appetitive-appetitive (*n*=6) (app–app) (**c**), appetitive–fear (*n*=4) (**d**) conditioned groups and in rats that received an unconditioned stimuli (*n*=6) (chocolate-foot shock, choco-FS) (**e**). Scale bar, 20 μm. (**f**) Percentages of cells expressing only *H1a* or *Arc* or both genes in the four behavioural groups. One-way ANOVA revealed differences among groups in the percentages of *H1a* (F_(3,17)_=65.84, *P*<0.001), *Arc* (F_(3,17)_=16.9, *P*<0.001), and doubly labelled cells (F_(3,17)_=10.93, *P*<0.001). Newman–Keuls test showed that appetitive–fear and unconditioned-treated animals did not differ from each other (*P*>0.05 in all instances, NS) while they were different from both the fear–fear and app-app groups. In the same way, the fear–fear and app-app groups did not differ from each other in all instances (*P*>0.05, NS). (**g**) Both appetitive–fear and unconditioned-treated animals had lower reactivation ratios than the fear–fear and app–app groups (F_(3,17)_=72.81, *P*<0.001). (**h**) In each group, the percentage of *H1a*-positive cells was similar to that of *Arc*-positive cells (fear–fear, *t*_8_= 0.55, NS; app-app, *t*_10_=1.37, NS; appetitive–fear, *t*_6_=−0.40, NS; unconditioned treated rats, *t*_10_=−0.24, NS). *P<0.05; **P<0.01, ***P<0.001; NS, not significant.

We then investigated the existence and characteristics of value-coding neurons in the lateral regions of the amygdala (Fig. 7a), an area that is involved in both fearful and hedonic experiences^2^,^3^,^37^ and is anatomically connected with the Te2 bidirectionally^13^,^14^,^15^. In the same rats in which we previously analysed Te2 neurons, the percentage of amygdalar neurons doubly labelled for *H1a* and *Arc* was significantly lower in the appetitive–fear conditioning group (Fig. 7d,f) than in the fear–fear (Fig. 7b,f) and appetitive–appetitive (Fig. 7c,f) conditioning groups. In contrast, the percentage of singly labelled neurons was significantly higher (Fig. 7d,f). Similarly, the reactivation ratio in the appetitive–fear conditioned animals was significantly lower than that of fear–fear conditioned rats (Fig. 7g). Thus, the lateral amygdala also has distinct neurons that respond either to pleasant or unpleasant stimuli.

Remarkably, the percentage of neurons singly labelled for *H1a* or *Arc* after the presentation of unconditioned incentive and aversive stimuli was higher than that observed in the fear–fear and appetitive–appetitive conditioned groups (Fig. 7e,f), whereas it was similar to that in the appetitive–fear conditioned animals (Fig. 7d,f). Similar results were obtained for the reactivation ratio (Fig. 7g). Finally, the percentages of *H1a*-positive and *Arc*-positive cells were also similar (Fig. 7h, Supplementary Fig. 10).

These data suggest that neuronal activity in the amygdala, unlike in Te2, is more broadly responsive to both innate and learned emotional processes. Remarkably, in both innate and conditioned processes, the two contrasting events activated similar percentages of neurons (Fig. 7f-h). This finding suggests that the lateral amygdala treats the two opposite valences in similar ways, in contrast with the common idea that lateral amygdala is mainly or more markedly involved in the analysis of threat stimuli.

### Te2 neurons targeted disruption impairs memory retention

So far, it is unknown if the observed activated neurons in Te2 play a *causal* role in maintaining learned emotional information. Therefore, we used a recently developed technique termed Daun02 inactivation^38^, which allows the selective manipulation of neurons engaged by a specific experience without affecting the surrounding cells. We used *c-fos-lacZ* transgenic rats carrying a transgene in which a *c-fos* promoter drives the transcription of the *lacZ* gene, resulting in the expression of β-galactosidase (β-gal). This induction only occurs in strongly activated neurons in which β-gal and Fos are coexpressed but not in the surrounding non-activated or weakly activated cells^39^. These activated neurons were inactivated, 90 min after rats performed a behavioural task, by administration of the prodrug Daun02: β-gal converts Daun02 into daunorubicin, which induces apoptotic cell death^39^. We used this model to determine if memory-activated cortical neurons are necessary for the storage of remote emotional memories.

**Figure 8:**
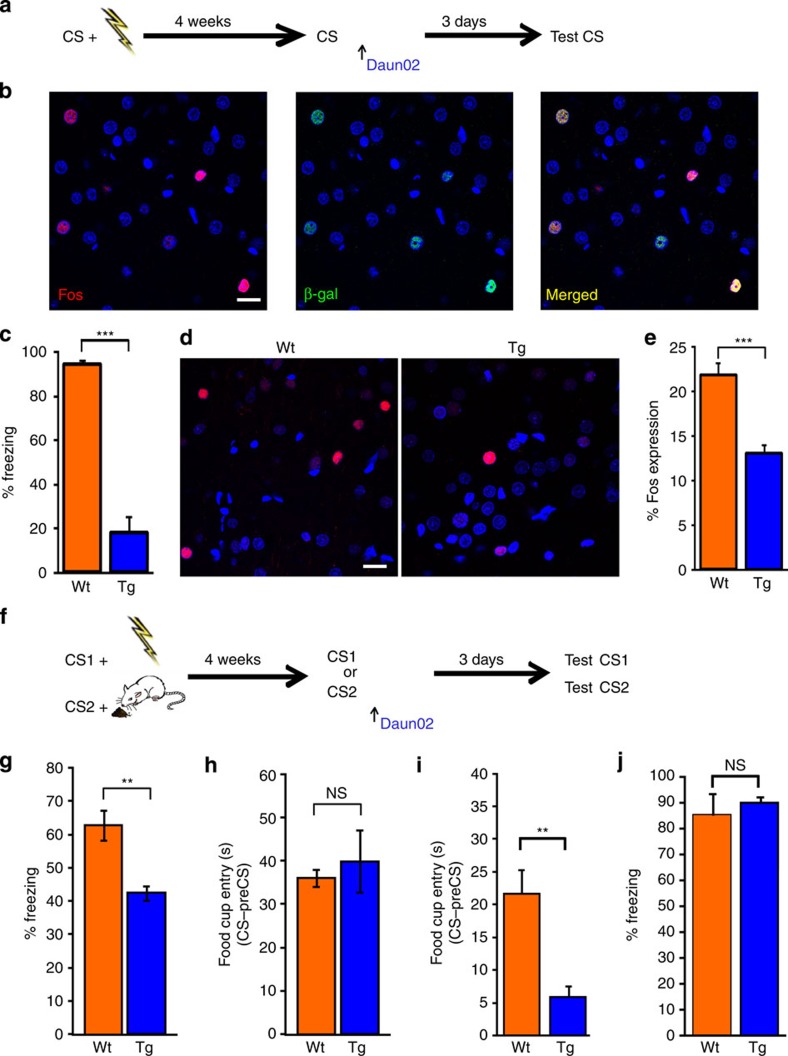
Fearful memory-activated cortical neurons are essential for the storage and retention of remote fear, but not incentive, memories. (**a**) Experimental design for Daun02 inactivation. *c-fos-lacZ* transgenic (Tg, *n*=6) and wild-type (Wt, *n*=5) rats were exposed to the association between a tone and an aversive stimulus. After 4 weeks, remote fear memories were retrieved by re-presenting the CS; 90 min later, Daun02 was injected into Te2. After 3 days, we measured the freezing response to CS. (**b**) Remote fear memory recall induced Fos expression (red nuclei) and β-galactosidase expression (green nuclei) in Te2 neurons. Merged panel shows nuclei doubly labeled for β-galactosidase and Fos (yellow). Scale bar, 20 μm. (**c**) Freezing response during CS administration in *c-fos-lacZ* rats was significantly less than that of Wt rats (*t*_9_= −8.75, *P*<0.001). (**d**) Photomicrographs of Fos staining in Te2 neurons from Wt (left) and *c-fos-lacZ* (right) rats 90 min after memory retrieval. (**e**) Fos-positive cell count was significantly higher in Wt rats than in *c-fos-lacZ* animals (*t*_9_= −5.42, *P*<0.001). (**f**) *c-fos-lacZ* and Wt rats were trained to associate two different auditory stimuli (CS1 and CS2) to opposite emotional experiences; 4 weeks later, remote fear memories were recalled by re-presenting CS1 or CS2, and 90 min later, Daun02 was injected in Te2. After 3 days, fear behavior and conditioned appetitive responses were measured by presenting CS1 and CS2. (**g**,**h**) In the groups where fear memories were recalled, freezing behavior (**g**) was significantly less in *c-fos-lacZ* rats (*n*=6) than in Wt rats (*n*=6) (*t*_10_= 4.20, *P*<0.01), whereas total time spent in the food cup during CS2 administration, minus a preCS period, (**h**) did not differ among groups (*t*_10_= −0.51, NS). (**i**,**j**) Conversely, in the groups where appetitive memories were recalled, appetitive memories (**i**) were impaired in *c-fos-lacZ* rats (*n*=8) compared to Wt rats (*n*=6) (*t*_12_= 4.21, P<0.01), while freezing behavior (**j**) did not differ among groups (*t*_12_= −1.55, NS). **P<0.01, ***P<0.001; NS, not significant. All data are mean and s.e.m.

Transgenic and wild-type rats were exposed to the association between a tone and an aversive stimulus, and 4 weeks later, fear remote memories were retrieved by re-presenting the CSs (Fig. 8a). In the transgenic rats, this procedure induced β-gal in 84.6±2.6% of the cortical neurons expressing Fos (Fig. 8b). Ninety minutes later, both transgenic and wild-type rats were infused with Daun02 in Te2 and then returned to their home cages for 3 days to allow cell-specific inactivation to occur^38^,^39^. On test day, transgenic animals showed amnesia to the CSs while wild-type rats did not (Fig. 8c). Compared with wild-type animals, transgenic rats had markedly fewer Fos-expressing neurons following the testing of fear memory retention (Fig. 8d,e). To rule out the possibility that the amnesia could be due to nonspecific neurotoxicity, we examined Nissl-stained sections of the Te2 cortex and found no gross alterations in the shape or size of the cortical architecture, nor any indication of gliosis (Supplementary Fig. 11), as previously reported^38^. These results suggest that cortical neurons activated by remote memory recall are necessary for the storage of remote emotional memories.

To further test the specificity of our manipulation, another group of transgenic and wild-type rats was exposed to associations between one tone and an aversive US and another tone and an appetitive US. Four weeks later, remote fear memories were retrieved by re-presenting the CS previously paired with the aversive US (Fig. 8f). Ninety minutes later, transgenic and wild-type rats were infused with Daun02 in Te2. After 3 days, transgenic animals showed amnesia but wild-type rats did not (Fig. 8g). Conversely, transgenic and wild-type rats had similar conditioned responses to the CS previously paired to appetitive CS (Fig. 8h), indicating that the observed amnesia is not due to nonspecific neurotoxicity in Te2. In a complementary experiment, transgenic animals showed amnesia to appetitive memories (Fig. 8i) but not to fear memories (Fig. 8j) when the Daun02 was injected after the reactivation of appetitive memories. Altogether, these results support the view that cells in Te2 activated by a specific emotional experience are needed for the encoding of its valence but not for the processing of auditory information and also suggest that different populations are necessary in Te2 for the two contrasting memories.

## Discussion

Collectively, our data lead to the idea that Te2 is necessary for the encoding and storage of the affective meaning that has been associated with sensory stimuli during an experience. Several alternative interpretations of our results can be ruled out. Te2 is not necessary for the encoding of the CS’s physical attributes. In fact, Te2 inactivation after pairing a neutral sound and an odour did not affect the subsequent remote memory of this association. Furthermore, the remote memory recall of two different tones activated a largely overlapping population of neurons in the fear–fear and appetitive–appetitive conditioning groups. This view is in accordance with previous findings^40^,^41^,^42^,^43^,^44^.

Moreover, we also ruled out that Te2 may serve as a key node for linking auditory stimuli to specific behavioural responses. These findings bear remarkable similarity to those of Pi *et al*.^45^ who showed that two different punishments (air puff and foot shock) generated similarly strong phasic activations of cortical neurons, suggesting that such neurons ‘signal the aversive quality of the negative feedback’^45^.

We also showed that the role of Te2 in emotional processes goes beyond the transmission of auditory CS to the amygdala. Inactivation of Te2 during early memory consolidation impaired remote, but not recent fear memories. This amnesia cannot be due to an impairment of the transmission of auditory information to the amygdala, given that amygdala impairment during memory consolidation also hampers recent memories^16^,^46^. Moreover, catFISH analysis revealed that two different sounds previously paired to stimuli of similar emotional valence recruited the same neuronal populations whereas the same tones recruited two distinct populations when paired with opposing emotional information. These results indicate that neurons are present within the Te2 whose activity is shaped by the two different emotional memories rather than by the two different CSs. Therefore, these neurons do not merely serve to send auditory information to the amygdala. Remarkably, selective inactivation of memory-activated cells, through Daun02 injection, revealed that the contribution of Te2 to remote memories arises from these different populations of cells.

In line with our findings, previous studies showed that some cortical interneurons in the auditory cortex are responsive not only to auditory CSs but also to painful USs^45^,^47^ and appetitive USs^45^, indicating that the activity of these neurons is modulated by emotional information. A similar suggestion came from studies showing that the emotional content provided by similar auditory stimuli, but paired to an aversive or appetitive US, shaped the plasticity of the auditory cortical receptive field in opposite ways^48^,^49^. Altogether, these results raise the intriguing possibility that learned emotional value is a property of stimulus representation in the sensory cortex.

In addition, we pointed out a remarkable difference between amygdala and Te2 participation in emotional memory processes; in the latter site, ‘value-coding’ neurons are mainly related to associative processes linking sensory cues to affective properties whereas unconditioned emotional stimuli by themselves do not engage these cells. Thus, Te2 activity reflects not mere emotional processes but rather the affective value that has been linked to sensory stimuli following emotional experiences. Moreover, our work adds the important notion that amygdalar neurons are not able to support the retention of appetitive and aversive remote memories in the absence of higher order sensory cortices. Remarkably, recent studies have shown that the amygdala is capable of promoting highly specific, associative and enduring changes in the auditory cortex^50^,^51^. Thus, the convergence of emotional and sensory information at the level of Te2 may lead to the activation of associative value-coding neurons, thus enabling this site to encode the learned value endowed with specific auditory stimuli. The existence of overlapping but distinct sets of neurons and the presence of neurons that respond to both affective features simultaneously may enable these sites to consider rewards and threats at the same time, a crucial issue in a continuously changing world. A dysfunction in these neurons or in their fine connectivity may affect the ability to differentiate between neutral and frightening stimuli, thus leading to generalized fear and anxiety disorders.

## Methods

### Animals

Male Wistar rats (age, 65–70 days; weight, 250–350 g) were employed in all experiments but those involving the blockade of Fos-activated neurons. For these latter experiments, we employed *c-fos-lacZ* transgenic rats (age, 65–70 days; 250–350 g) that had been bred for 35–40 generations on a Sprague–Dawley background. Animals were housed in plastic cages with food and water available ad libitum, under a 12-h light/dark cycle (lights on at 0700 hours) at a constant temperature of 22±1 °C. All the experiments were conducted in accordance with the European Communities Council Directive 2010/63/EU and approved by the Italian Ministry of Health (authorization no. 265/2011).

### Stereotaxic surgery for Te2 and Oc2L treatments

Stereotaxic coordinates for Te2 and lateral Oc2 (Oc2L) lesions were taken from Paxinos and Watson^52^ with cortical fields referenced to Zilles^53^. Two pairs of injection sites were planned bilaterally using the following coordinates: for Te2, (i) AP=5.8, ML=±6.7, DV= 6.0 and (ii) AP=6.8, ML=±6.7, DV= 6.0; for Oc2L, (i) AP=5.8, ML=±6.7, DV=4.0, and (ii) AP=6.8, ML=±6.5, DV=4.0. A burr hole, permitting the penetration of a 28 gauge needle, was drilled over each injection site. The needle was connected to a 10 μl Hamilton syringe connected to an infusion pump.

Reversible inactivation in Te2 and Oc2L were made with tetrodotoxin (TTX; 10 ng μl^−1^ in physiological saline; Tocris Bioscience, catalog no. 10789), or with muscimol (1 mg ml^−1^ in physiological saline) (Tocris Bioscience, catalog no. 0289). A 0.5 μl volume of TTX, muscimol, or vehicle (control animals) was injected, and the needle was left in place for 1 min. Given that the effects of muscimol terminate within a few hours after injection^18^, we prolonged its action by injecting it on two consecutive days. For the analysis of muscimol diffusion, we used muscimol conjugated to fluorescent BODIPY TMR-X (Life Technologies, catalog no. M23400), at 0.5 mg ml^−1^ in saline.

Protein kinase Mzeta inhibition was achieved by injecting the cell-permeable zeta inhibitory peptide (ZIP)^30^,^31^. ZIP (Tocris Bioscience, catalog no. 2549) and its inactive scrambled control (scr-ZIP; QCB, 20-6210) were prepared in saline at 10 nmol μl^−1^; 0.5 μl was used per injection site.

For the treatment of *c-fos-lacZ* transgenic rats, Daun02 (Hycultec, catalog no. HY-13061) was dissolved to a final concentration of 5 mg ml^−1^ in a solution of 10% DMSO, 6% Tween-80, and 84% PBS. A volume of 0.7 μl was used per injection site.

Irreversible lesions in Te2 and Oc2L were made by injecting NMDA (Tocris Bioscience, catalog no. 0114). NMDA was dissolved in phosphate-buffered saline, pH 7.4, to a concentration of 20 μg μl^−1^, and 0.20 μl was injected. The needle was left in place after the injection for 3 min.

After treatment, the incision was closed with stainless steel wound clips, and the animal was given a subcutaneous injection of the analgesic/anti-inflammatory ketoprofen (2 mg kg^−1^ body weight); it was kept warm and under observation until recovery from anaesthesia.

### First-order fear conditioning

Rats were trained to associate a sensory stimulus with a foot shock in an conditioning chamber^12^ (Coulbourn Instruments). The floor was made of stainless steel rods connected to a shock generator set to deliver 0.7 mA current. The chamber was fitted with a loudspeaker connected to a tone generator set to deliver an 80 dB, 1,000 Hz pure tone (auditory CS), and a 12 W fluorescent light bulb (rise and decay times, 100 ms; visual CS); both the loudspeaker and light bulb were located 20 cm above the floor. One animal at a time was placed inside the chamber and left undisturbed for 2 min. Then, it was exposed to a series of seven consecutive auditory or visual CSs, each lasting 8 s and paired, during the last 1 s (for auditory conditioning) or 2 s (for visual conditioning), respectively, with an electric foot shock; the seven sensory stimuli were separated by intervals of 22 s.

### Tests of first-order fear memory retention

Retention of fear memory was tested in apparatuses different from those used for conditioning, and in a different room, to avoid conditioned fear behaviour to contextual cues^12^.

Freezing responses were tested in a transparent plastic cage enclosed within a sound-attenuating box equipped with an exhaust fan, which eliminated odourized air from the enclosure and provided background noise of 60 dB. Before testing, animals were handled for 5 min on 2 consecutive days. On the third day, they were placed in the testing chamber and left undisturbed for 2 min. Then, CSs were administered in a manner identical to that used during conditioning, but without the foot shock. The rats’ behaviour was recorded by a digital video camera and the videos were reviewed to determine the duration of the freezing response, taken as an index of fear. Freezing response was expressed as the percentage of time during, which there was complete absence of somatic mobility, except for respiratory movements. The assessment of freezing was performed by one person blinded to the animal’s assignment to an experimental group.

Avoidance responses were tested in a two-chamber light–dark box consisting of a lighted chamber of white opaque plastic with a transparent lid and a similarly sized dark chamber with walls and ceiling of dark opaque plastic. The floors of both chambers were plastic. Between the two chambers there was a rectangular opening (6 × 8 cm). The lighted chamber was illuminated to 60 lux and equipped with a food cup for the delivery of sucrose pellets. Before testing, rats were food restricted to 80–90% of their free-feeding weight, and they were habituated to the apparatus for 4 days (20 min per day). To test memory retention, an animal was placed in the lighted chamber and exposed to the series of seven tones, as described above. Avoidance response was expressed as the percentage of time spent in the dark chamber after the first tone.

### Sensory preconditioning

CS1 consisted of almond odour presented using a flow-dilution olfactometer. Clean air (1.5 l min^−1^) was directed to a solenoid valve that, when operated, passed the air to a 15-ml bottle containing 10 ml of almond essential oil. Odourized air was then directed to the conditioning chamber via ¼-inch Tygon tubing for an exposure duration of 8 s. CS2 was the same tone (80 dB, 1,000 Hz, 8 s) used in first-order fear conditioning. Two different chambers, which differed in olfactory, visual and tactile features, were used in the experiment.

On each of the first 2 days, animals spent 20 min in each of the two different chambers used in the experiment, in the absence of auditory or olfactory stimuli. On day 3, the animals were returned to one of the chambers and, after a 3-min adaptation period, were preexposed, over 20 min, to six sensory stimuli (3 CS1 and 3 CS2) presented in a pseudorandom order, avoiding that the same CS be presented more than two times consecutively. The interval between the end of one CS and the start of the next was 2 min.

On day 4, the animals were returned to the same chamber for sensory preconditioning. After a 4-min adaptation period, they were exposed to the two stimuli in a paired or unpaired fashion. A groups of animals received the two stimuli paired, in five cycles each consisting of CS2 immediately followed by CS1 and then a 40-s pause. Control rats received the stimuli unpaired, that is, separated by intervals of about 6 min; this control served to rule out stimulus generalization, namely the tendency for a response to generalize from one stimulus to another^24^. All animals remained in the chamber for 2 min after the final stimulus. Then, animals were rapidly transferred to the stereotaxic apparatus for Te2 inactivation with TTX, muscimol or vehicle (performed within 10–15 min). On day 5, the animals received no behavioural manipulation. On days 6 and 7, the animals were placed in the conditioning chamber (the one with the grid floor) and were exposed to CS1 in association with the aversive US (foot shock with 0.7 mA current during the last 1 s of each CS). CS1–US pairing was presented nine times at intervals of 42 s.

Remote memories were tested in two new and different chambers. Animals were habituated to the new environments on days 27 and 28 (5 min per day). Then, they underwent one CS2 test session on day 29 and one CS1 test session on day 30. Rats were placed into one of the chambers and, after a 3-min adaptation period, were exposed to the stimulus eight times at intervals of 42 s. The freezing response was measured as described above.

### Second-order fear conditioning

The overall protocol for second-order fear conditioning was similar to that used with sensory preconditioning. The preexposure step (days 1–3) was identical. On day 4, animals were placed in the conditioning chamber and exposed to CS1 in association with the aversive US: a foot shock (0.7 mA current) was given during the last 1 s of each CS1 (CS1–US pairing). On day 5, rats underwent a second session of CS1–US pairing. On day 6, animals were exposed to the two sensory stimuli, paired exactly as on day 4 in the sensory preconditioning protocol above; Te2 inactivation with TTX, muscimol or vehicle was also performed as in that step. On day 7, no behavioural manipulation was performed. The retention of remote memories was tested on days 27–30 as described for sensory preconditioning.

### Appetitive-to-fear conditioning

Rats were placed on a restricted diet to maintain their body weight at approximately 80–90% of their free-feeding weight. One day before training, rats were given ∼1 g of chocolate-flavoured sucrose pellets (Bio-Serv, catalog no. F07256) in their home cages to familiarize them with the pellets. For training, rats were placed in a conditioning chamber without a grid floor, enclosed in a sound-attenuating box. They were trained to associate the auditory CS (80 dB, 1,000 Hz) with an appetitive US, namely one chocolate-flavoured pellet delivered at the end of the tone into the food cup. The CS–US pairing was presented 28 times with a variable interval during a 60 min session, once per day for four consecutive days.

Four weeks later, rats were placed in a different environment and exposed to the same CS, this time paired with an aversive US (0.5 mA) in the absence of the food reward. The CS–US pairing was repeated 7 times over 10 min. Memory retention was tested 1 week later. Briefly, animals were placed in the testing chamber for 2 min (preCS period) and then exposed to the CS seven times (8 s tone followed by 17 s pause). Retention of memories was measured on both the freezing response (conditioned aversive response) and the conditioned discriminative response. Conditioned discriminative responses were calculated as the extra time (in s) an animal spent with its head in the chamber’s food cup, searching for a reward in response to the CS, according to the formula: (time in food cup during the CS (8 s) and post-CS presentation (17 s) minus the 25 s preceding CS onset.

### First-order appetitive conditioning

Rats were trained to associate a pleasant US (chocolate-flavoured food pellet) with an auditory, olfactory or visual CS, essentially as described for appetitive-to-fear conditioning.

### Conditioning protocols prior to catFISH analyses

Training sessions were conducted in distinct chambers differing in olfactory, visual and tactile features and located in different rooms. The CS1 was a pure tone (8 s, 80 dB, 1,000 Hz), while the CS2 was an 11-s train of frequency-modulated upsweeps (500-ms duration, logarithmically modulated between 5 and 15 kHz, 50ms rise and fall, 78 dB). The US was an electric foot shock of 0.18 mA or, to elicit a stronger (for example, more salient) aversive memory, 0.25 mA current, or a chocolate-flavoured food pellet. Conditioning to CS1 was performed in the conditioning chamber as described earlier for first-order fear conditioning. Conditioning to CS2 was performed in a plastic cage composed by four black Plexiglas sheets, with a grid floor.

Fear–fear conditioning (Supplementary Fig. 6c) involved six sessions composed of three aversive trainings per CS. A series of six auditory CSs was administered (except for the last two sessions of the fear–fear conditioning in which only 3 CSs paired with USs per session were presented). The last 1 s of each CS was paired with an aversive US.

Appetitive–appetitive conditioning consisted of 13 sessions (9 CS1 and 4 CS2 appetitive trials). Each appetitive trial was performed as described above (see also Supplementary Fig. 6d).

Appetitive–fear conditioning (Supplementary Fig. 6e) consisted of 13 sessions with 9 CS1 (appetitive) and 4 CS2 (aversive) trials. Appetitive training with a pleasant US was conducted as described above in ‘First-order appetitive conditioning’. Rats were also conditioned to an aversive CS2 as described in the fear–fear conditioning protocol above.

Retention of remote memories was tested 4 weeks after the start of the training. Rats were handled for three consecutive days (5 min per day) and habituated to two unfamiliar cages. CS1 conditioned responses were tested by presenting CS1 four times (8 s, with a 22 s interval). The session lasted 2 min after which the rat was returned to its home cage for 20 min. Then, the animal was placed in a different environment and exposed to CS2 four times (11 s, with a 19-s interval). The conditioned appetitive response was calculated as described above in the ‘Appetitive-to-fear conditioning’. The freezing response was also measured.

### Chocolate–footshock unconditioned stimulation

Two days before the test, rats were placed on a restricted diet to maintain their body weight at approximately 90% of their free-feeding weight. In addition, animals were habituated to being transported outside their animal room to a new room. A day before the behavioural protocol began, rats were given ∼1 g of pleasant (chocolate-flavored) food pellets in the home cage to familiarize them with those pellets. The following day, rats received ∼1 g of the pellets in their home cages. Twenty minutes later, they were transported to a Skinner box. Immediately afterwards, rats were subjected to seven footshocks (1 s, 0.18 mA) at 1-s intervals. At the end of the stimulation, rats were returned to their cages, having spent a total time of ≈15 s inside the conditioning apparatus. This procedure allowed administering USs in such a short time as to prevent the association of the temporally compressed unconditioned stimuli to the surroundings^35^,^54^.

### Fluorescent *in situ* hybridization (catFISH)

catFISH analysis was used to examine the expression of *Arc/Arg 3.1* (*Arc*) and *Homer 1a* (*H1a*) genes. Briefly, an *Arc* antisense riboprobe was directed to the region from exon I to III, while an *H1a* probe was directed to the 3′UTR. The vectors were linearized with EcoR1, purified and used for *in vitro* transcription with the DIG RNA Labeling kit (SP6/T7) (Roche, 11175025910), in the presence of fluorescein-UTP (incorporated into the *H1a* probe) or digoxigenin-UTP (incorporated into the *Arc* probe). The yield and integrity of riboprobes was confirmed by gel electrophoresis. At the end of this process, probes were purified by spin chromatography.

To detect *Arc* expression, mounted sections were incubated with digoxigenin-labelled *Arc* riboprobe followed by anti-digoxigenin–POD (1:500, Roche, 11207733910) and a cyanine-3 substrate kit (1:50, NEL744001KT, PerkinElmer). After detection of the *Arc* riboprobe, the slides were treated with 2% H_2_O_2_ to quench residual POD activity. Fluorescein-labelled *H1a* probe was detected with anti-fluorescein-POD (1:500, Roche, 11426346910) and a fluorescein substrate kit (1:50, NEL741001KT, PerkinElmer). Nuclei were counterstained with a mounting media containing DAPI (Vector, H1200). The specificity of the labelling was confirmed by omitting the riboprobes.

Slides were imaged using a Leica SP5 confocal microscope using three lasers (488, 520 and 570 nm) corresponding to peaks in the emission spectra of DAPI (cell nuclei), fluorescein (*H1a* mRNA) and Cy3 (*Arc* mRNA), respectively. The objective lens was set at × 63 magnification. Data were acquired using a *z*-stack (1-μm thickness per section in a stack), the height of which was determined by the penetration of one detectable probe per sample (usually 8-μm thickness per stack). The pinhole, photomultiplier tube gain and contrast settings were constant for all image stacks acquired from a slide. Cells were considered for analysis if the nucleus was present in at least 4 sections of the z-stack. Only putative neurons were included in the analysis, and glial cells, identified from their small size (∼5 μm diameter) and bright, uniform nuclear counterstaining, were excluded. Cells that were positive for both DAPI and Cy3 were considered *Arc*-positive, cells with both DAPI and fluorescein were considered *H1a*-positive, and cells with DAPI, Cy3 and fluorescein were positive for both mRNA.

Cells counts were performed manually; to prevent bias, the experimenter was blinded to the relationship between the images and the behavioural conditions they represented. Raw data were expressed as a percentage of the total neuronal nuclei analysed per stack. Typically, 16 confocal z-stacks (175 × 175 μm square; zoom fraction, 1.4) were taken from each animal: images were collected from four bilateral slides at two different anteroposterior coordinates (−6.5 mm and −7.0 mm from the bregma)^52^,^53^. Due to the extension of Te2 cortex, two z-stacks were collected in every slide. In the lateral amygdala, 12 confocal z-stacks (189 × 189 μm square; zoom fraction, 1.3) were taken from each animal, taking six bilateral samples at two anteroposterior coordinates (−2.6 mm and −3.0 mm from the bregma)^52^,^53^. The percentages of stained cells for different groups were presented as mean and s.e.m. In addition, the reactivation ratio was calculated as the percentage of doubly labelled cells divided by the percentage of *H1a*-positive cells^33^.

### Tissue preparation and histological procedures

See Supplementary Notes.

### Statistical analyses

Concerning catFISH analysis, raw data were expressed as a number of neurons expressing *Arc*, *H1a* or both genes divided for the total neuronal nuclei analysed per stack. The percentages of stained cells for different groups were presented as mean (and s.e.m.). A two-tailed Student’s *t*-test was used for experiments with two groups, while for experiments with more than two groups we used one-way ANOVA followed by Newman–Keuls multiple comparisons test.

## Additional information

**How to cite this article:** Grosso, A. *et al*. The higher order auditory cortex is involved in the assignment of affective value to sensory stimuli. *Nat. Commun.* 6:8886 doi: 10.1038/ncomms9886 (2015).

## Supplementary Material

Supplementary Information Supplementary Figures 1-11, Supplementary Note 1 and Supplementary References
