# Supplementary material for: The higher order auditory cortex is involved in the assignment of affective value to sensory stimuli
Source: Nat Commun. 2015 Dec 1;6:8886. doi: 10.1038/ncomms9886 (PMC5482717; doi:10.1038/ncomms9886)
Supplement: Supplementary Information — Supplementary Figures 1-11, Supplementary Note 1 and Supplementary References [file ncomms9886-s1.pdf]

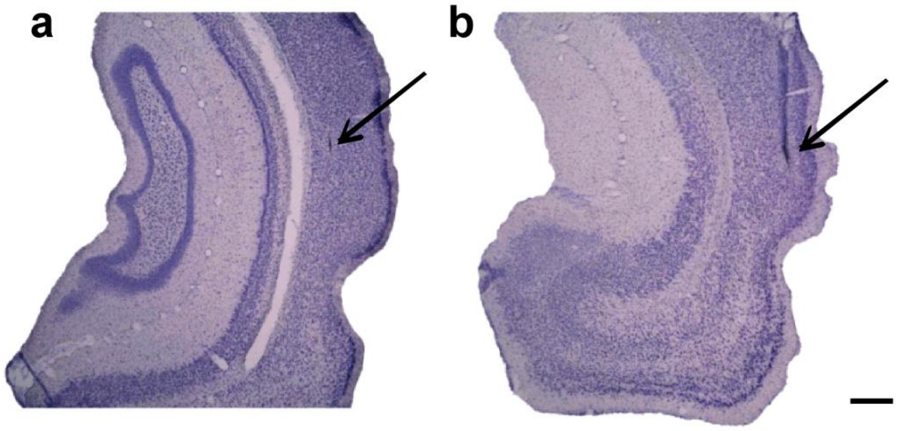

Supplementary Figure 1. Representative photomicrographs of Nissl staining from animals injected with tetrodotoxin (TTX) into Te2. The arrow indicates the position of the needle track. Scale bar, 500 µm.

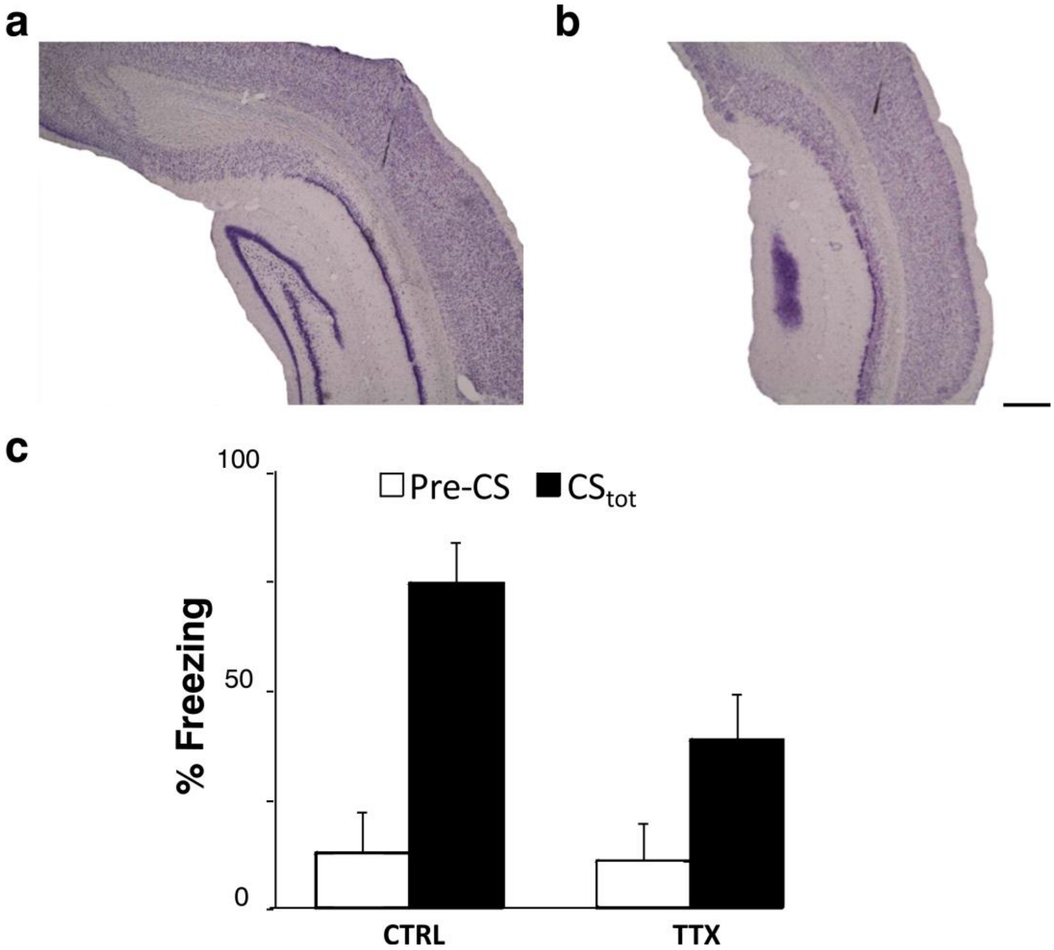

Supplementary Figure 2. Reversible inactivation of secondary visual cortex impairs remote fearful memory to visual CSs. (a, b) Histological localization of the needle track in the secondary visual cortex Oc2L around the stereotaxic coordinates AP = - 5.8 (**a**) and AP = - 6.8 (**b**) in TTX-injected animals. Scale bar, 450 µm. (**c**) Freezing response to visual CSs was significantly lower in TTX-injected (n=8) animals than in control animals (CTRL, n=8) (t_{14}=8.21, P < 0.001). All data are mean and SEM.

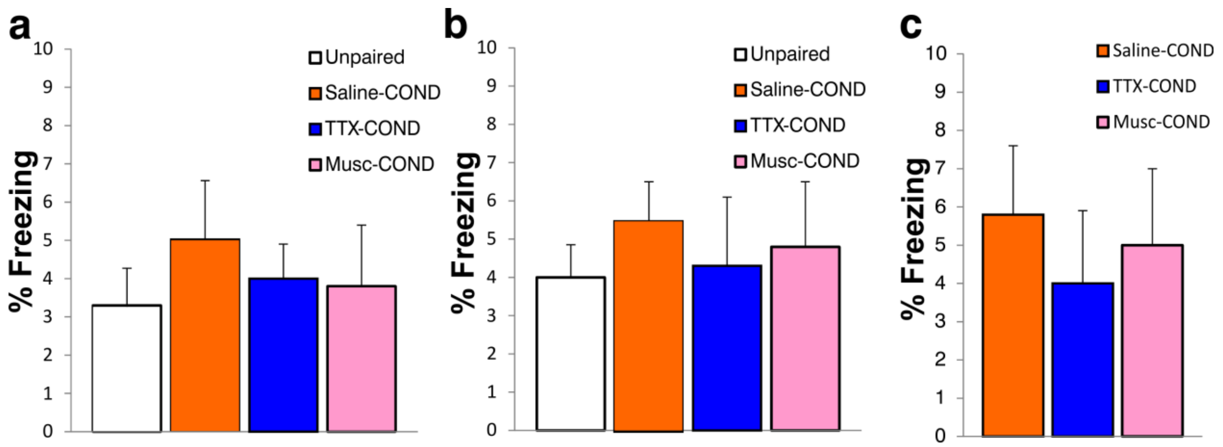

Supplementary Figure 3. Absence of generalization in TTX-, Muscimol- and Saline-injected rats. Freezing response expressed as percentage of time in the first 2-min period in the new context before CS presentation during sensory preconditioning (**a**), second-order conditioning (**b**) and appetitive-to-fear conditioning (**c**). One-way ANOVA did not show statistical differences in all instances: sensory preconditioning ($F_{(3,33)}$= 0.31, n.s.), second order conditioning ($F_{(3,33)}$= 0.22, n.s.) and appetitive to fear paradigm ($F_{(2,24)}$= 0.24, n.s.). All data are mean and SEM.

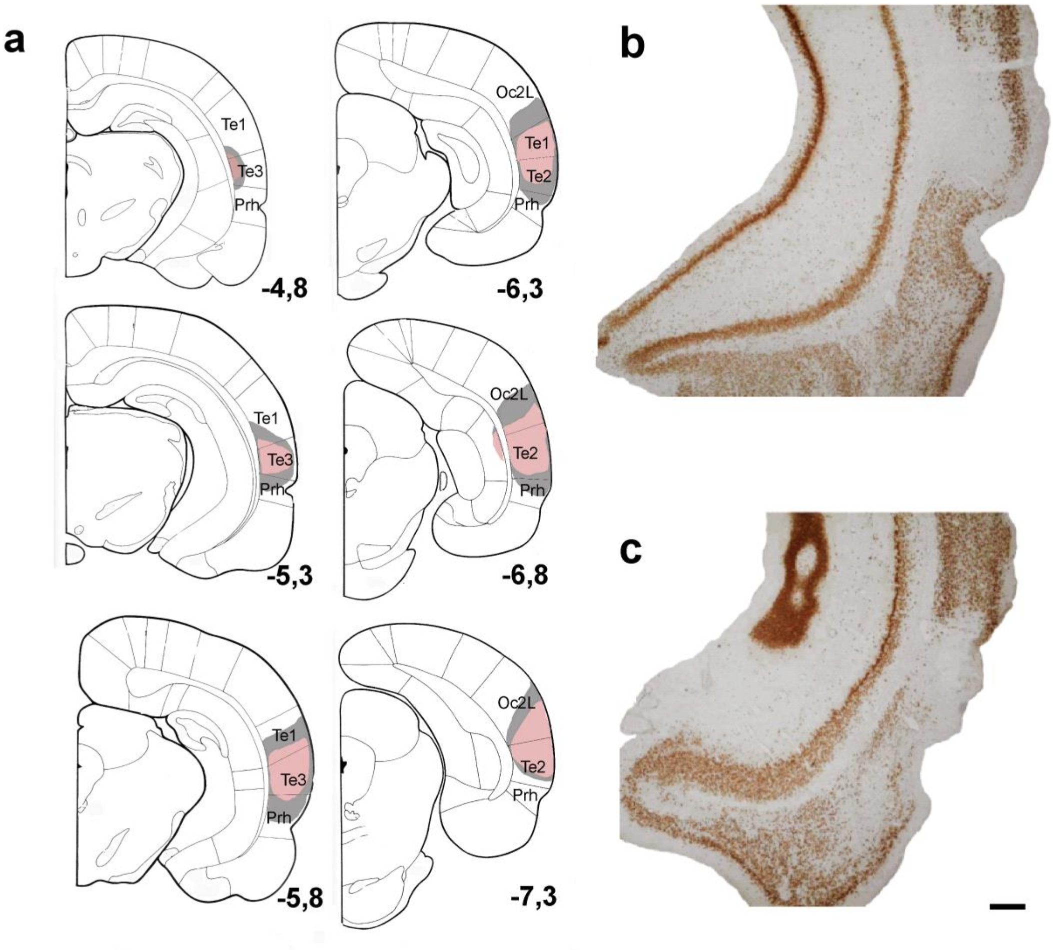

Supplementary Figure 4. Extension of NMDA-induced excitotoxic lesions of Te2. (**a**) Histological reconstruction of the excitotoxic lesions aimed at the Te2 area. Pink and gray areas represent the smallest and largest extent of the lesions, respectively. Negative numbers indicate posterior distance from the bregma. Plates adapted from the atlas of Zilles[2]. Oc2L, secondary occipital visual cortex; PRh, perirhinal cortex; Te1, Te2 and Te3, primary, secondary and tertiary auditory cortex. (**b, c**) Coronal sections through Te2 cortex stained with NeuN antibody. Scale bar, 500 µm.

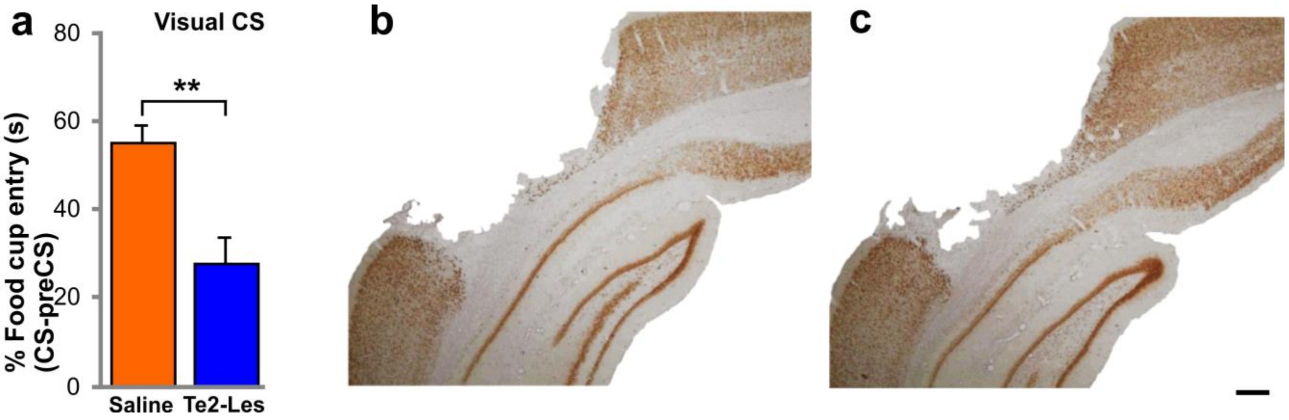

Supplementary Figure 5. Appetitive long-term memories to visual CSs are hampered by secondary visual cortex lesions. (**a**) Total time spent in the food cup during CS administration minus a preCS period in conditioned sham-operated (n = 9) and Oc2-lesioned (n = 8) rats. Student's t-test showed that Oc2-lesioned animals differed from the sham-operated conditioned rats (t_{15}= 3.69, P < 0.01). (**b, c**) NeuN staining of Oc2 cortical damage. Scale bar, 500 µm. * P < 0.05; ** P < 0.01, *** P < 0.001; ns, not significant. All data are mean and SEM.

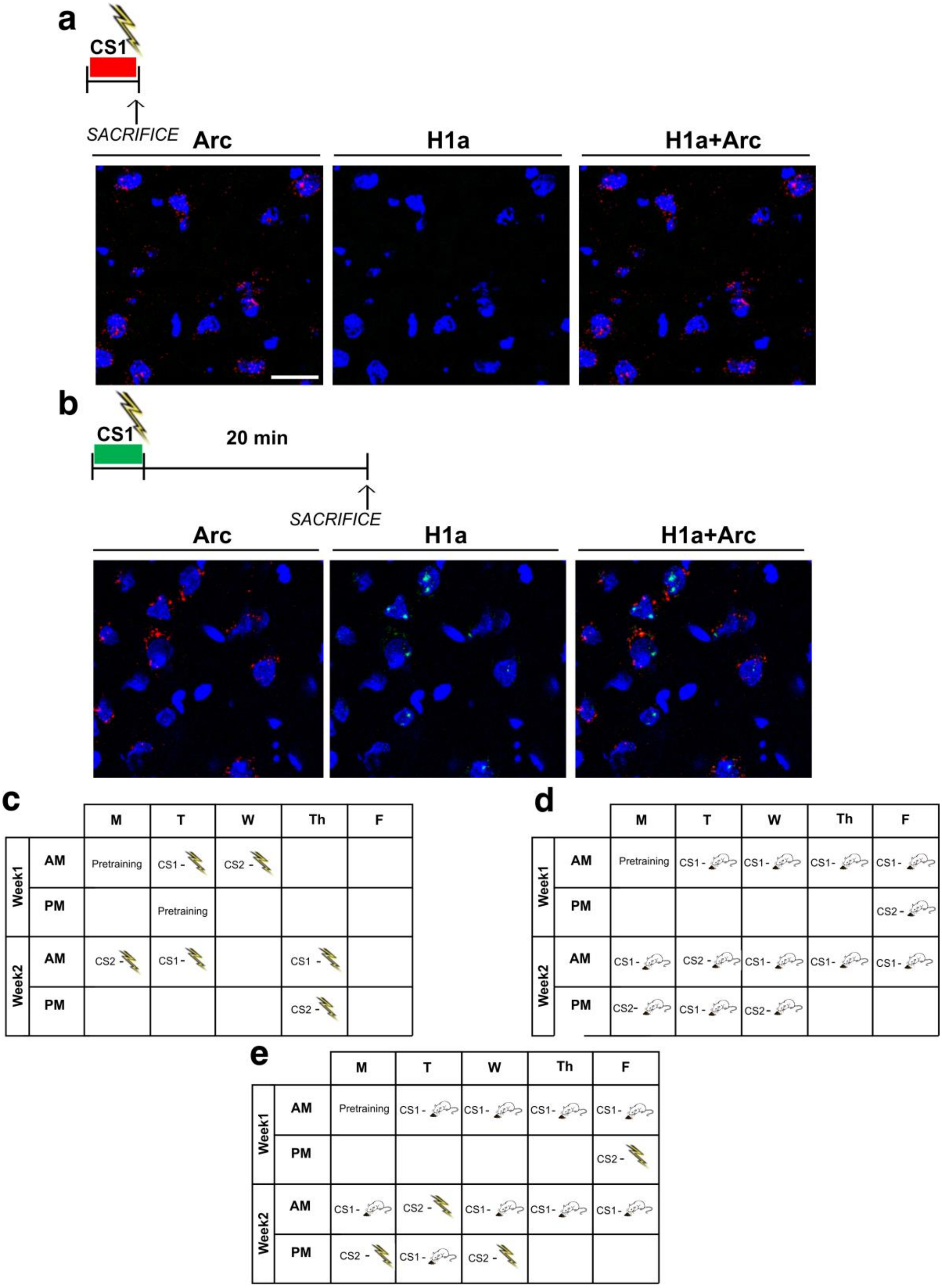

Supplementary Figure 6. Distinct temporal profiles of *Arc* and *H1a* RNA detected by catFISH. (a, b) Time-dependent expression of *Arc* and *H1a* mRNA following testing for fear memory retention. Rats were killed immediately or 20 minutes after the test. Immediately after memory recall, there is a rapid expression of *Arc* in the nucleus (**a**) while *H1a* starts to appear in the

nucleus only 25 minutes after the session. At this time interval, *Arc* is already detectable in the cytoplasm. (**b**) These data confirms the functionality of probes. Scale bar, 20 µm. (**c**) In the fear-fear conditioning group, two different acoustic stimuli (CS1 and CS2) were paired with an aversive foot shock (US) in order to get similar fearful valences. (**d**) In the appetitive-appetitive conditioning (app-app) group, CS1 and CS2 were paired with the delivery of pleasant US (food) in order to get similar incentive valences. (**e**) In the appetitive-fear conditioning, CS1 and CS2 were paired with an appetitive (chocolate) and aversive (foot shock) US, respectively. The behavioral training consisted of 13 sessions (9 appetitive and 4 aversive). Monday, M; Tuesday, T, Wednesday, W; Thursday, Th; Friday, F.

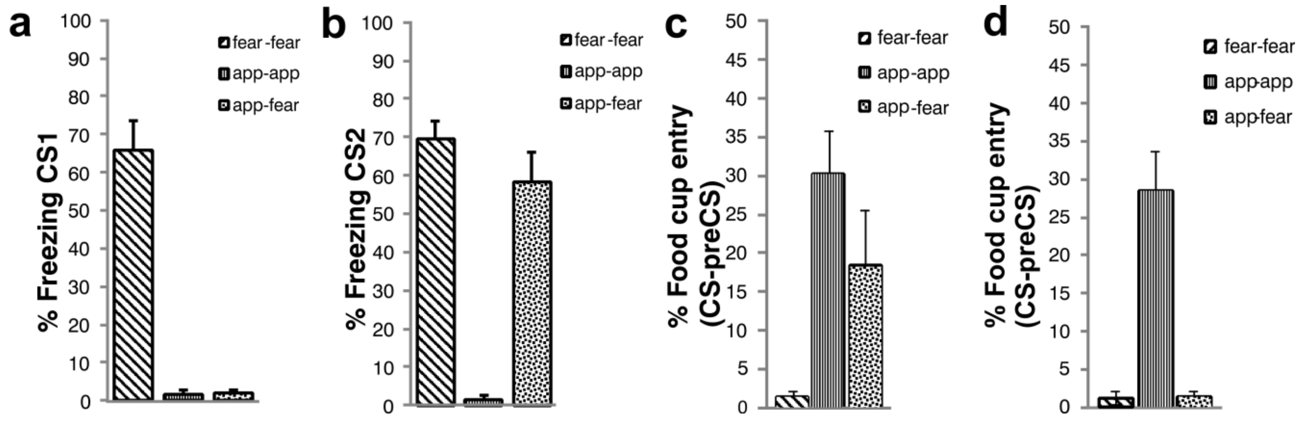

Supplementary Figure 7. Behavioral responses following the retrieval of emotional memories. (**a, b**) Percentage of freezing in fear-fear (n = 9), appetitive-appetitive (app-app) (n = 6) and appetitive-fear (n = 8) conditioning groups. (**a**) Freezing response to CS1 was higher in the fear-fear conditioning group than in the app-app and appetitive-fear conditioning groups. (**b**) In contrast, the freezing response to CS2 was significantly higher in both fear-fear and appetitive-fear conditioning groups than in the app-app conditioning group. (**c**) Total time spent in the food cup during CS1 minus a preCS period was significantly higher in both app-app and appetitive-fear groups than in the fear-fear group. (**d**) The appetitive response to CS2 was higher in the app-app group than in the fear-fear and appetitive-fear conditioned animals. All data are mean and SEM.

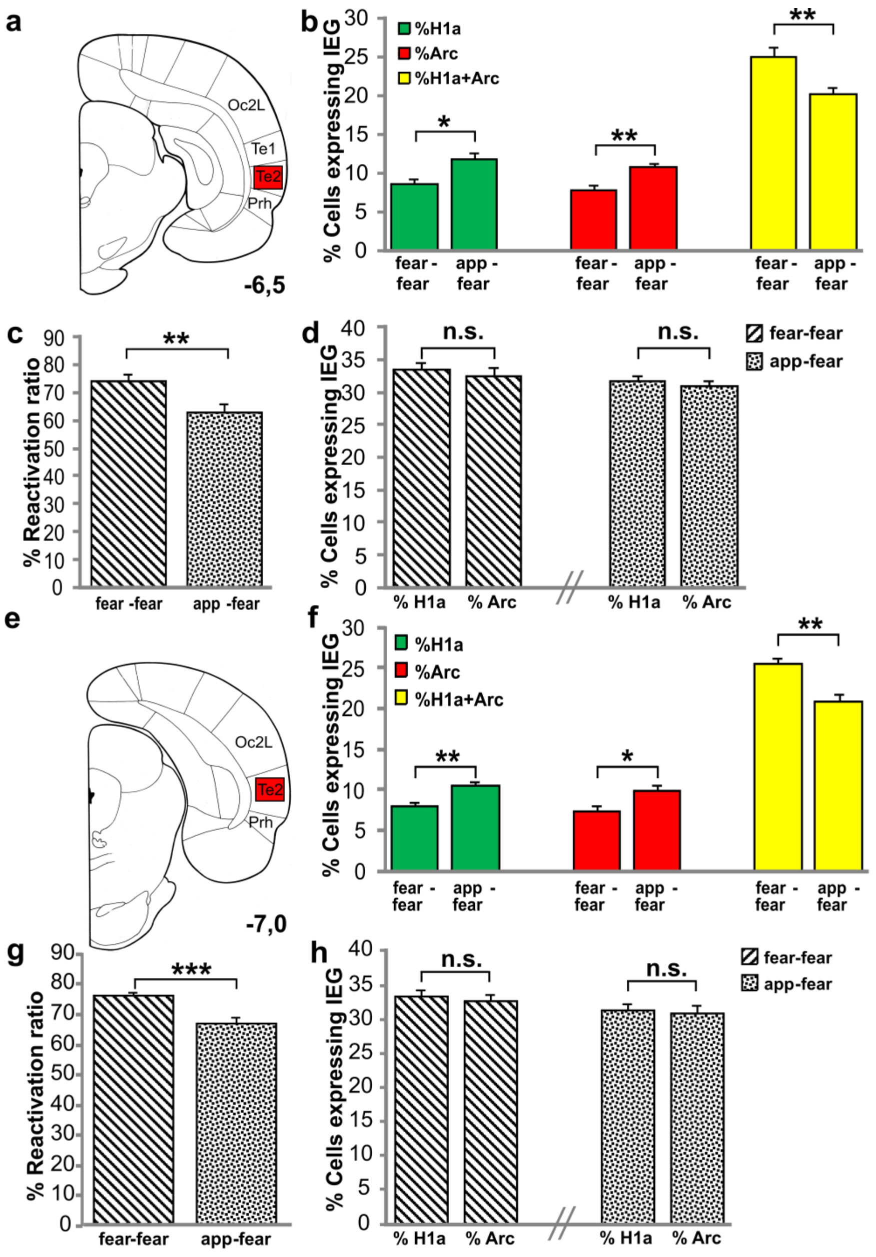

Supplementary Figure 8. Te2 neuronal activity following the recall of emotional memories measured at two different cortical sections. (**a, e**) Schematic representation of the two separate regions of the Te2 cortex: anteroposterior, AP = -6.5 mm (**a**) and -7.0 mm (**e**) from the bregma. Plates adapted from the atlas of Zilles[2]. Red boxes indicate the position of the area analyzed. Oc2L, secondary occipital visual cortex; PRh, perirhinal cortex; Te1 and Te2, primary and secondary auditory cortex. (**b-d**) Te2 neuronal activity in the region around AP = -6.5 mm from the bregma. (**b**) Percentages of cells expressing immediately early genes (*H1a*, *Arc*, and doubly labeled cells) in the two behavioral groups (AP= -6.5 mm). The percentages of neurons singly labeled for *H1a* (t_{15}=

2.93, $P < 0.05$) and *Arc* (t_{15}= 3.40, $P < 0.01$) mRNA were significantly higher in the appetitive-fear group than in the fear-fear conditioned group, while the percentage of doubly labeled cells was less (t_{15}= -2.99, $P < 0.01$). (**c**) In the appetitive-fear conditioned group, the reactivation ratio was significantly less than that in the fear-fear conditioned group (t_{15}= -3.33, $P < 0.01$). (**d**) In each behavioral group, the percentage of *H1a*-positive cells was similar to that of *Arc*-positive cells (fear-fear, t_{16}= -0.49, ns; appetitive-fear, t_{14}= -0.84, ns). (**f-h**) Te2 neuronal activity in the area corresponding to AP= -7.0 mm. (**f**) The percentage of neurons singly labeled for *H1a* (t_{15}= 3.42, $P < 0.01$) and for *Arc* (t_{15}= 2.88, $P < 0.05$) mRNA was statistically higher in the appetitive-fear group than in the fear-fear conditioned group, while the percentage of doubly labeled cells was less (t_{15}= -3.82, $P < 0.01$). (**g**) In the appetitive-fear group, the reactivation ratio was significantly less than that in the fear-fear conditioned group (t_{15}= -4.49, $P < 0.001$). (**h**) In each group, the percentage of *H1a*-positive cells was similar to that of *Arc*-positive cells (fear-fear, t_{16}= -0.45, ns; appetitive-fear, t_{14}= -0.35, ns). * $P < 0.05$; ** $P < 0.01$, *** $P < 0.001$; ns, not significant. All data are mean and SEM.

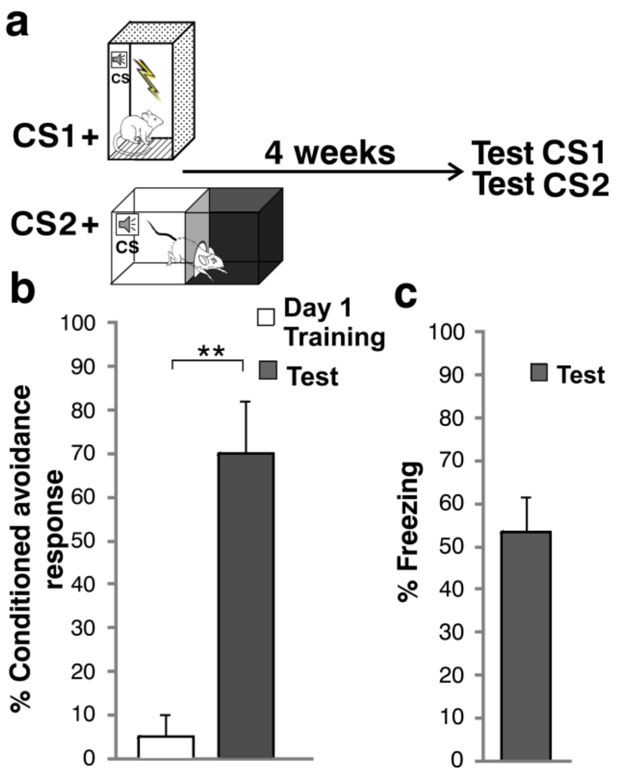

Supplementary Figure 9. Te2 codes the aversive valence of memory rather than the different behavioral response. (**a**) Experimental design illustrating the avoidance-freezing experiment. Rats were trained to associate one auditory CS (CS1) to escapable foot shocks, so as to induce a learned escape avoidance behavior while another CS (CS2) was paired to an inescapable foot shock so as to induce freezing behavior. During long-term memory retention trials, CS1 induced escape responses whereas CS2 elicited freezing. (**b**) In the avoidance-fear group (n=5), the percentage of conditioned avoidance response to CS1 was calculated during the first day of training (Day 1) and during the test day. One-way ANOVA with repeated measures revealed a significant difference in the percentage of avoidance between the first day of training and the test day ($F_{(1,4)}= 42.25$, $P < 0.01$). (**c**) Fear behavior to CS2, measured as the freezing response. ** $P < 0.01$. All data are mean and SEM.

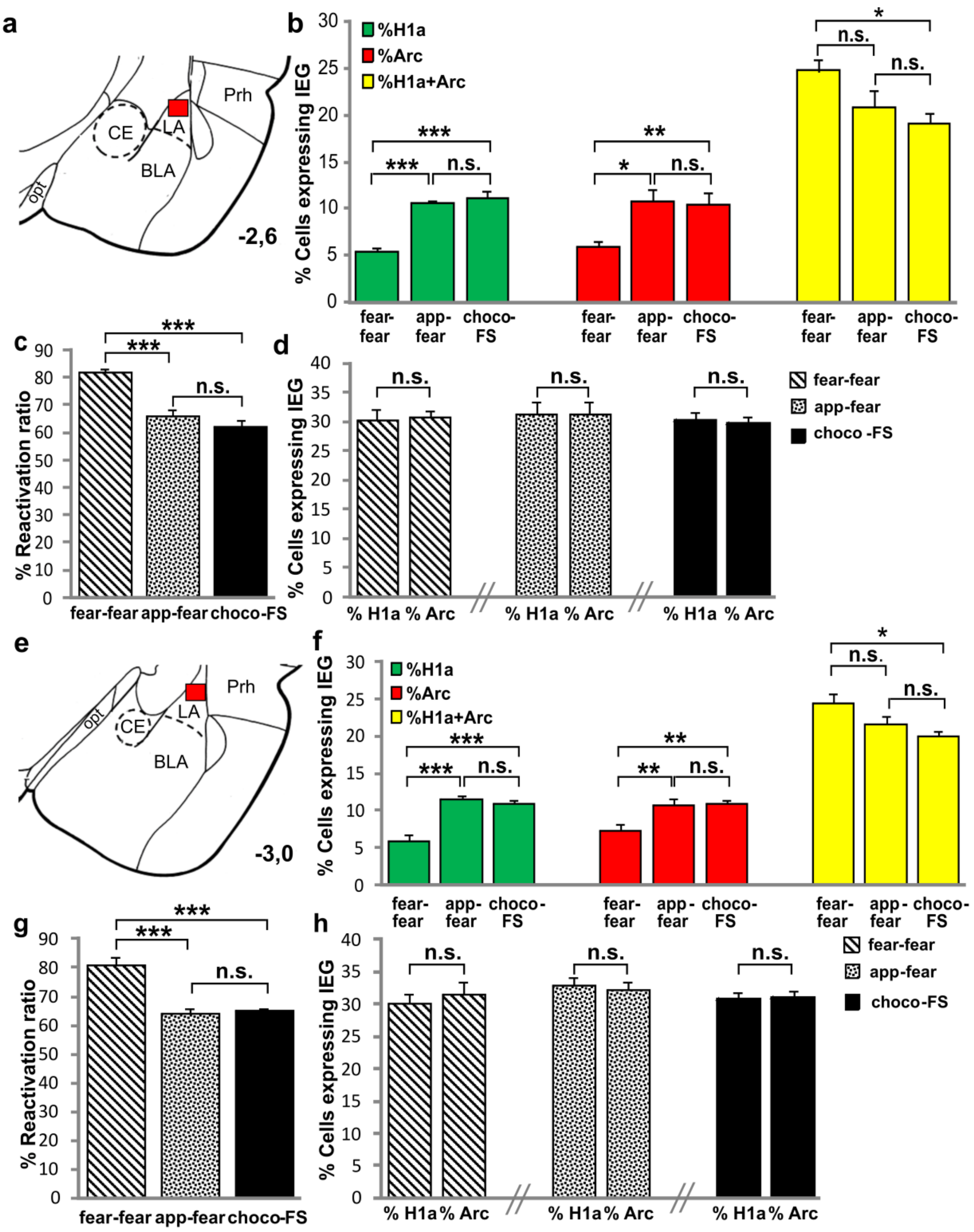

Supplementary Figure 10. Lateral amygdala neuronal activity following emotional memory recall in two anatomically distinct areas. (**a, e**) catFISH analysis was performed at two separate regions of the lateral amygdala: anteroposterior, AP = -2.6 mm (**a**) and -3.0 mm (**e**) from the bregma. Plates are adapted from Zilles[2]. Red boxes indicate the position of the magnified images of the dorsolateral part of the lateral amygdala. BLA, basal lateral amygdala; CE, central amygdala; LA, lateral amygdala; Opt, optic tract; PRh, perirhinal cortex. (**b-d**) LA activity in the region around AP= -2.6 mm from the bregma. (**b**) Percentages of cells expressing immediately early genes

(IEG) (*H1a*, *Arc* and doubly labeled cells) in the three behavioral groups: fear-fear, appetitive-fear, chocolate-foot shock (choco-FS). One-way ANOVA revealed differences among groups in the percentage of *H1a* ($F_{(2,12)}$ = 34.36 P < 0.001), *Arc* ($F_{(2,12)}$ = 8.95, P < 0.01) and doubly labeled cells ($F_{(2,12)}$ = 4.31, P < 0.05). (**c**) Both the appetitive-fear and the unconditioned-treated animals had a lower reactivation ratio than the fear-fear group ($F_{(2,12)}$ = 32,15, P < 0.001). (**d**) In each group, the percentage of *H1a*-positive cells was similar to that of *Arc*-positive cells (fear-fear, t_8= 0.81, ns; appetitive-fear, t_6= 0,015, ns; unconditioned treated rats, t_{10}= -0.37, ns). (**f-h**) LA activity in the area corresponding to AP= -3.0 mm from bregma. (**f**) One-way ANOVA revealed differences among groups in the percentages of *H1a* ($F_{(2,12)}$ = 21.38, P < 0.001), *Arc* ($F_{(2,12)}$ = 9.45, P < 0.01) and doubly labeled cells ($F_{(2,12)}$ = 4.16, P < 0.05). (**g**) Both the appetitive-fear and the unconditioned-treated animals had a lower reactivation ratio than the fear-fear group ($F_{(2,12)}$ = 23.76, P < 0.001). (**h**) In each group, the percentage of *H1a*-positive cells was similar to that of *Arc*-positive cells (fear-fear, t_8= 0.61, ns; appetitive-fear, t_6= -0.50, ns; unconditioned treated rats, t10= 0.13, ns). * P < 0.05; ** P < 0.01, *** P < 0.001; ns, not significant. All data are mean and SEM.

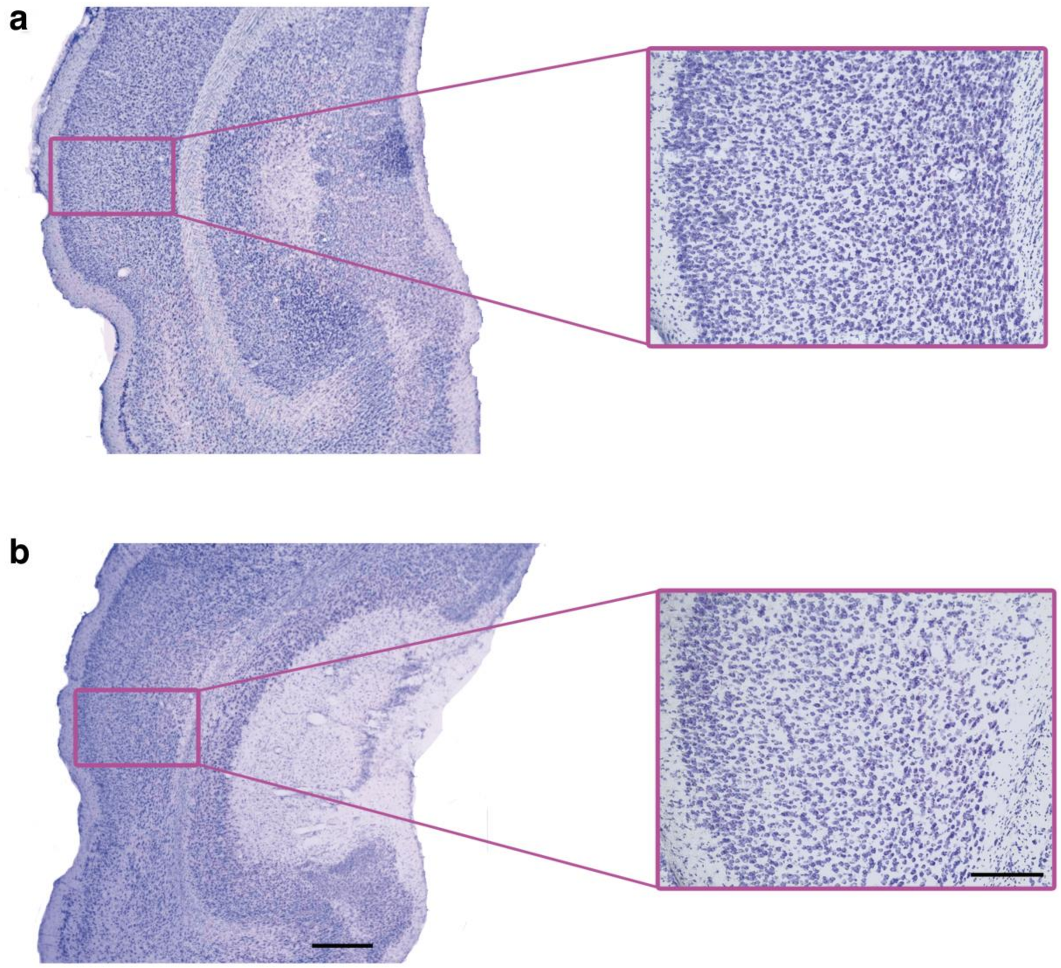

Supplementary Figure 11. Daun02 did not produce generalized damage in Te2. Representative photomicrographs of Nissl staining obtained 3 days after treatment with Daun02 in wild-type rats (**a**) and c-fos-lacZ rats (**b**). Left, scale bar, 500 µm. Right, scale bar, 250 µm. Gross alterations of Te2 architecture or gliosis was not seen at either magnification.

Supplementary Methods

Tissue preparation and histological procedures

Immediately after testing, rats were placed in an airtight plastic container saturated with isoflurane vapor and then rapidly decapitated with a guillotine. Brains were quickly removed and frozen in isopentane that had been supercooled on dry ice (approximately -80°C). Frozen brains were stored at -80°C. For sectioning, brains were warmed to -20°C and fixed to the platform of a cryostat with Tissue-TeK O.C.T. Compound (VWR). Sections (20 μm thick) were mounted on slides (Superfrost Plus, VWR), which were then sealed in boxes and stored at -80°C until use.

Beta-galactosidase and Fos immunohistochemistry with *cfos-lacZ* rats

On the induction day, 90 minutes after memory testing, a group of *cfos-lacZ* rats was deeply anaesthetized and perfused intracardially with 4% paraformaldehyde in order to examine the colocalization of Fos and β-galactosidase expression. The brains were dissected, stored overnight at 4 °C, and transferred to 30% sucrose. Coronal sections (50 μm) were cut on a vibratome and collected in phosphate-buffered saline (PBS). Free-floating sections were incubated in a blocking solution (4 % bovine serum albumin (BSA), 10 % normal goat serum and 0.5 % Triton X-100) for 1 h at room temperature. Then, they were incubated in rabbit antibody to c-Fos (1:500 dilution, Santa Cruz Biotechnology, sc-52) and sheep antibody to β-gal (1:1000, Aves Labs, BGL-1010) in the blocking solution overnight at 4 °C. Subsequently, sections were washed with PBS and incubated for 1 h at room temperature with AlexaFluor 488-labeled goat anti-sheep IgG (1:400 dilution, Life Technologies, A11039) and AlexaFluor-568-labeled goat anti-rabbit IgG (1:400 dilution, Life Rechnologies, A11036) diluted in PBS, for 1 h on a shaker at room temperature. Sections were washed in PBS, mounted with mounting media containing DAPI (Vector, H1200) and cover-slipped.

Fos immunohistochemistry.

On 'test day', ninety minutes after the completion of memory retention test both wild-type and *fos-lacZ* rats were deeply anaesthetized and perfused intracardially with 4% PAF. Brain sections were processed for Fos immunohistochemistry. We used the primary antibody to c-Fos (1:500, Santa Cruz Biotechnology, sc-52) and the sections were developed with AlexaFluor-568-labeled goat anti-rabbit antibody (1:400 dilution, Lifetechnologies, A11036) as described previously.

Immonuhistochemistry analysis. Tissues were imaged using three lasers (488, 520 and 570 nm), each corresponding this time to the peak emission spectrum for DAPI (Nissl stain for cell nuclei), Fluorescein *(β-gal)* and CY3 (*Fos),* respectively. Data were acquired using a *z*-stack (1 µm thickness per section in stack), the height of which was 4 µm. Cells were counted for analysis if the nucleus was present on at least 3 sections of the z-stack. The objective lens was set at 63× magnification. Cells which were positive for both DAPI and CY3 were considered Fos-*positive*, cells with both DAPI and fluorescein were considered β-gal-positive, and cells with DAPI, CY3 and Fluorescein were considered double-labeled for both proteins. The results were expressed as a percentage of the total neuronal nuclei analyzed per stack.

Typically, 8 confocal z-stacks (175x175 µm square area; zoom fraction=1,4) were taken from each animal: images were collected from 4 slides at antero-posterior distance of -6,5 mm from the Bregma[1,2]. Due to the extension of Te2 cortex, in every slide were collected two z-stacks.

Histological procedures

In the reversible inactivation procedure, needle track placement was verified with Nissl staining. The extension of the damaged areas in the case of NMDA lesions was histologically verified at the end of the experiments with NeuN staining analysis (**Supplementary Fig. 3**). Verification of lesions was made observing NeuN-stained tissue under a microscope magnified at 2x and 4x. The lack of staining was used as an indication of neuronal loss due to lesions. The spread of fluorescent

muscimol was determined by observing mounted slides in a stereomicroscope (Leica, MZ16FA) equipped with a fluorescent lamp (Hg, 100 W) and a digital camera (Leica, DFC360FX).

Supplementary References

1. Paxinos, G. & Watson, C. *The Rat Brain in Stereotaxic Coordinates* (New York: Academic Press Elsevier) (1986).

2. Zilles, K. *The Cortex of the Rat* (Berlin: Springer-Verlag) (1985).
